# ω-3 PUFA for Secondary Prevention of White Matter Lesions and Neuronal Integrity Breakdown in Older Adults: A Randomized Clinical Trial

**DOI:** 10.1001/jamanetworkopen.2024.26872

**Published:** 2024-08-01

**Authors:** Lynne H. Shinto, Charles F. Murchison, Lisa C. Silbert, Hiroko H. Dodge, David Lahna, William Rooney, Jeffrey Kaye, Joseph F. Quinn, Gene L. Bowman

**Affiliations:** 1 NIA-Layton Aging and Alzheimer’s Disease Center, Department of Neurology, Oregon Health & Science University, Portland; 2 Department of Biostatistics, University of Alabama, Birmingham; 3 Portland VA, Portland, Oregon; 4 Interdisciplinary Brain Center, Department of Neurology, Massachusetts General Hospital and Harvard Medical School, Boston; 5 Advanced Imaging Research Center, Oregon Health & Science University, Portland; 6 Parkinson’s Disease Center, Oregon Health & Science University, Portland; 7 McCance Center for Brain Health, Department of Neurology, Massachusetts General Hospital and Harvard Medical School, Boston

## Abstract

**Importance:**

Older adults with lower intake and tissue levels of long-chain ω-3 polyunsaturated fatty acids (PUFAs) eicosapentaenoic acid (EPA; 20:5) and docosahexaenoic acid (DHA; 22:6) have more brain white matter lesions (WMLs), an association suggesting that small-vessel ischemic disease, a major contributor to the development of dementia, including Alzheimer disease, may be preventable through ω-3 treatment.

**Objective:**

To determine whether ω-3 treatment reduces WML accumulation in older adults without dementia harboring WMLs and with suboptimal ω-3 status.

**Design, Setting, and Participants:**

This quadruple-blinded, placebo-controlled, randomized clinical trial with treatment stratification by apolipoprotein E ε4 allele (APOE*E4) carrier status used linear mixed-effects models to estimate mean annual change between groups. The study was conducted at Oregon Health & Science University, a major academic medical center in the Pacific Northwest, from May 2014 to final participant visit in September 2019. Data analysis concluded in July 2022. Participants were adults without dementia aged 75 years and older with WMLs greater than or equal to 5 cm^3^ and plasma ω-3 PUFA less than 5.5 weight percentage of total.

**Intervention:**

Three-year treatment with 1.65 g of ω-3 PUFA (975 mg of EPA and 650 mg of DHA) vs a soybean oil placebo matched for taste, smell, and appearance.

**Main Outcomes and Measures:**

The primary outcome was annual WML progression measured using magnetic resonance imaging. Secondary outcomes included diffusion tensor imaging of fractional anisotropy (DTI-FA), representing neuronal integrity breakdown.

**Results:**

A total of 102 participants (62 women [60.8%]; mean age, 81 years [range, 75-96 years]) were equally randomized, 51 per treatment group. Although the ω-3 group had less annual WML accumulation than the placebo group, the difference was not statistically significant (1.19 cm^3^ [95% CI, 0.64-1.74 cm^3^] vs 1.34 cm^3^ [95% CI, 0.80-1.88 cm^3^]; *P* = .30). Similarly, the ω-3 group had less annual DTI-FA decline than the placebo group, but the difference was not statistically significant (−0.0014 mm^2^/s [95% CI, −0.0027 to 0.0002 mm^2^/s] vs −0.0027 mm^2^/s [95% CI, −0.0041 to −0.0014 mm^2^/s]; *P* = .07). Among APOE*E4 carriers, the annual DTI-FA decline was significantly lower in the group treated with ω-3 than the placebo group (−0.0016 mm^2^/s [95% CI, −0.0032 to 0.0020 mm^2^/s] vs −0.0047 mm^2^/s [95% CI, −0.0067 to −0.0025 mm^2^/s]; *P* = .04). Adverse events were similar between treatment groups.

**Conclusions and Relevance:**

In this 3-year randomized clinical trial, ω-3 treatment was safe and well-tolerated but failed to reach significant reductions in WML accumulation or neuronal integrity breakdown among all participants, which may be attributable to sample size limitations. However, neuronal integrity breakdown was reduced by ω-3 treatment in APOE*E4 carriers, suggesting that this treatment may be beneficial for this specific group.

**Trial Registration:**

ClinicalTrials.gov Identifier: NCT01953705

## Key Points

**Question:**

Does ω-3 polyunsaturated fatty acid treatment reduce cerebral white matter lesion (WML) accumulation and neuronal integrity breakdown among older adults?

**Findings:**

In this randomized clinical trial of 102 participants, ω-3 treatment failed to significantly reduce WML progression and neuronal integrity breakdown among all participants; however, apolipoprotein E ε4 allele (APOE*E4) carriers who received ω-3 had significant reductions in neuronal integrity breakdown over 3 years.

**Meaning:**

Although ω-3 treatment failed to reach significant reduction in WML progression and neuronal integrity breakdown among all participants at risk for dementia, the findings suggest that APOE*E4 carriers may benefit from ω-3 treatment.

## Teaser

This randomized clinical trial examines whether treatment with ω-3 polyunsaturated fatty acids (PUFAs) reduces white matter lesion accumulation in older adults without dementia who have white matter lesions and suboptimal ω-3 status.

## Introduction

Cerebral white matter lesion (WML) accumulation, identified as hyperintensities on magnetic resonance imaging (MRI) fluid-attenuated inversion recovery (FLAIR) scans, are thought to play a major role in the development of cognitive decline and dementia, including Alzheimer disease (AD).^1,2^ WMLs are associated with risk for cognitive decline and AD, and cerebral amyloid angiopathy and arteriosclerosis coexist among approximately 80% of probable AD cases examined at autopsy.^3,4,5,6,7^ The WML pathophysiological mechanisms involve endothelial cell activation and blood-brain barrier breakdown, cerebral hypoperfusion, and reduced regenerative capacity of oligodendrocytes.^8,9,10,11^ The confluent WMLs that abut the ventricles and span deeper into the subcortical tissue may be more representative of astrogliosis, axonal degeneration, and a progressive myelin loss, whereas isolated periventricular WMLs may be more indicative of microglial activation as a neuroinflammatory response to peripheral stimuli through a disturbed blood-brain barrier.^12,13,14^ Whole-brain diffusion tensor imaging of fractional anisotropy (DTI-FA) is an indicator of white matter microstructural integrity associated with cognitive function that declines with aging, particularly among apolipoprotein E ε4 allele (APOE*E4) carriers.^15,16,17^

Diet-derived bioactive lipids, such as the ω-3 polyunsaturated fatty acids (PUFAs) eicosapentaenoic acid (EPA; 20:5) and docosahexaenoic acid (DHA; 22:6), which are found in deep, cold-water fish,^18^ and the plasma concentrations that manifest from their consumption are associated with reduced WML burden.^19,20^ These ω-3 PUFAs are modifiable structural phospholipids that may alter cell signaling and metabolism toward a less inflammatory state by acting as substrate for synthesis of lipophilic inflammation-resolving metabolites^21^; ω-3 has been shown to reduce microglial cytokine release (ie, tumor necrosis factor–-α) and endothelial and immune cell surface protein CD54 expression.^22,23^ CD54 protein encoding inferred by the intercellular adhesion molecule-1 (ICAM-1) gene locus can increase blood-brain barrier permeability.^24^ Meta-analyses of ω-3 clinical trials have shown that it can downregulate soluble adhesion molecules, specifically ICAM-1, although other ω-3 trials have identified modest effects on cerebral perfusion and white matter integrity parameters.^22,25,26^ Plasma ω-3 explains 24% of the WML variation, and higher levels are associated with a 40% reduction in probability for WML among older adults.^19,20^ Plasma ω-3 concentrations above 11.0 mg/dL (to convert to millimoles per liter, multiply by 0.0355) are associated with less WML-mediated executive cognitive function decline in older adults without dementia,^27^ and 1.65 g per day of ω-3 has been shown clinically to supersede this hypothetical neuroprotective threshold in mild-to-moderate AD. Consequently, we targeted enrollment to a population of older adults harboring WMLs and suboptimal ω-3 status to determine whether ω-3 treatment prevents WML progression and neuronal integrity breakdown over 3 years vs a placebo.

## Methods

### Study Design and Participant Enrollment

The Polyunsaturated Fatty Acids for the Prevention of Cerebral Small Vessel Disease and Inflammation in Aging (PUFA trial) protocol and baseline results are available elsewhere.^28^ The trial protocol and statistical analysis plan are shown in Supplement 1. Briefly, this 3-year, quadruple-blinded, placebo-controlled, randomized clinical trial was conducted at Oregon Health & Science University (OHSU) and compared ω-3 vs placebo treatment (1:1) in participants recruited from the Portland, Oregon, metropolitan area. Eligibility requirements included age (≥75 years) and being cognitively intact or with mild cognitive impairment, defined as Clinical Dementia Rating (CDR) of 0.5 or less (range, 0.0-0.5, with lower scores denoting cognitively and functionally intact) and Mini-Mental State Examination (MMSE) score above 23 (range, 24-30, with higher scores denoting better cognitive function). Brain MRI screen used a 1-mm isotropic FLAIR algorithm to ascertain total WML eligibility of 5 cm^3^ or more. Plasma or whole-blood spot ω-3 eligibility excluded candidates with objective evidence of ω-3 status already above the threshold associated with neuroprotection (≥11.0 mg/dL or ≥5.5 weight percentage of total fatty acids) (eTable 1 in Supplement 2).^27^ Race and ethnicity were self-reported by the participants and are included in this study to reflect the generalizability of the results.

### Standard Protocol Approval and Patient Consent

This randomized clinical trial follows the Consolidated Standards of Reporting Trials (CONSORT) and Good Clinical Practice guidelines, with local ethical committee approval (OHSU Institutional Review Board). All participants signed written informed consent. A designated independent Data and Safety Monitoring Board was established and met biannually to review progress.

### Randomization and Masking

Eligible consented participants were randomized by an independent statistician (C.F.M.) using a modified minimization algorithm to ω-3 or placebo soft gels balanced by age across 5-year block segments, sex, education (up to high school graduate, at least some college, and baccalaureate graduate or postgraduate), screening total WML volume, and CDR scores.^29^ Participants, study investigators, research staff, and health care practitioners were masked to treatment assignments. Study ω-3 and placebo soft gels were independently received, processed, and distributed by OHSU research pharmacy staff. Blinding integrity was assessed with a short questionnaire completed by the participants and the investigators.^30^

### Procedures and Intervention

As described previously,^28^ eligibility was determined over 2 sequential clinic visits lasting 5 hours. MMSE, CDR, geriatric depression scale, safety laboratory (eg, comprehensive metabolic panel and complete blood counts), and ω-3 status (plasma or whole-blood spot) were determined at visit 1. Screening MRI for WML eligibility was at visit 2. Participants underwent a general and neurological examination, neuropsychological testing, safety laboratory workup, and other blood draws systematically across the trial visits.^28^ Treatment compliance was recorded through pill counts at each clinic visit, and telephone contact every 3 months between the clinic visits was performed to review compliance. Pill counts less than 80% adherent triggered further contact to resolve compliance. Mass-mailers and subsequent telephone screenings of 1100 people identified 299 candidates for invitation to an in-clinic assessment of general and neurological signs and symptoms, cognition, blood ω-3 status, and WMLs. The first clinic visit including ω-3 status yielded 233 candidates for WML MRI screening. There were 124 of the 233 who failed to meet WML screening eligibility (53%), and another individual with poor blood pressure management, which left 108 eligible individuals. The active group received 3 soft gels daily yielding 1.65 g of ω-3 (975 mg of EPA and 675 mg of DHA) in a triacylglycerol emulsion, whereas the placebo group received 3 soybean oil soft gels daily indistinguishable from active treatment according to taste, appearance, smell and texture by the manufacturer (Nordic Naturals, Inc). Random batches of the natural product underwent systematic independent laboratory testing over the course of the trial to ensure product integrity (eTable 2 in Supplement 2).

### Main Outcomes

The primary outcome was annual WML progression. Secondary outcomes were DTI-FA, medial temporal lobe gray matter (GM), and total brain GM. Treatment stratification by *APOE* genotype was prespecified. Baseline brain MRI was captured using a 3-T MRI instrument (Total Imaging Matrix Trio; Siemens) with phased array body transmit radiofrequency (RF) coil and a 32-channel head RF coil receiver housed in the OHSU Advanced Imaging Research Center. The Total Imaging Matrix Trio scanner was upgraded to a 3-T Prisma (Siemens) during the study. The RF receiver coil and sequence parameters were consistent across the upgrade, and the Prisma system was used for 32 of the 87 participants with a 1-year follow-up visit and each scan from there forward. Anatomical imaging sequences were similar to the Alzheimer Disease Neuroimaging Initiative protocols.^31^ Structural MRI included T_1_ magnetization prepared rapid gradient echo (MPRAGE), FLAIR, pseudo-continuous arterial spin labeling, DTI, proton density–T_2_, and functional MRI with the imaging session requiring approximately 60 minutes in total: 3-dimensional (3D) T_1_-MPRAGE (orientation, axial; repetition time, 2300 ms; echo time, 3.45 ms; slice thickness, 1 mm; slices, 144; native resolution, 1 mm isotropic; acquisition matrix, 256 × 192), 3D FLAIR (orientation, sagittal; repetition time, 6000 ms; echo time, 388 ms; slice thickness, 1 mm; slices, 160; native resolution, 0.5 × 0.5 × 1 mm; acquisition matrix, 512 × 512), and DTI (orientation, axial; repetition time, 9100 ms; echo time, 88 ms; native resolution, 2 mm isotropic; slice thickness, 2 mm; slices, 72; acquisition matrix, 128 × 128; b = 0, 1000 seconds/mm^2^). Briefly, GM volume of the medial temporal lobe, total brain, and ventricular cerebral spinal fluid volumes were segmented with FreeSurfer software version 6.0 (Harvard University), visually inspected, and manually corrected for errors. For FLAIR-derived WMLs, a semiautomated intensity-based thresholding algorithm using resampled 1 mm isotropic resolution images was used.^28,32^ FLAIR images were linearly coregistered to T1 images, and the mean FLAIR signal intensity in the WM was calculated. Voxel clusters at least 2.5 SD above the mean WM FLAIR signal intensity were used as seeds. The mean signal intensity of each cluster was calculated and compared with all surrounding voxels. Adjacent voxels of at least 95% of the cluster mean signal intensity were added to the cluster, and the process was repeated iteratively. The final WML mask was visually inspected and manually corrected before final volume calculation. After study completion, updated neuroimaging processing algorithms included analysis of higher resolution sequences (FLAIR, in-plane resolution 0.5 mm). Mean skeletal DTI-FA and mean, radial, and axial diffusivity were calculated using tract-based spatial statistics.^33^ Cognitive changes were not powered adequately but collected rather comprehensively to power future efforts (eAppendix in Supplement 2).

### ω-3 PUFA and *APOE* Genotype

Screening for study inclusion required either Holman Omega-3 whole-blood spot test EPA plus DHA less than 5.5% total fatty acids, which allows for a 10-day result turnaround, or plasma EPA plus DHA less than 11.0 mg/dL, which requires 14- to 20-day turnaround.^34,35^ Plasma fatty acid concentrations were analyzed by gas chromatography with flame ionization detection as previously described.^36^ Fatty acids were identified by comparison with a standard mixture of fatty acids (GLC OQ-A; NuCheck Prep), which was also used to determine individual fatty acid calibration curves. The C23:0 TG was used to calculate recovery efficiency of the assay and applied to all fatty acids. Plasma fatty acids were expressed as absolute concentrations (eg, micrograms per milliliter of plasma). *APOE* genotype was determined by polymerase chain reaction test.

### Safety

Safety evaluation included general and neurological examination, heart rate and blood pressure, use of concomitant medications and dietary supplements, and blood laboratory testing (ie, complete blood counts, comprehensive metabolic panel, prothrombin time, and international normalized ratio) performed at screening and at 6, 12, 24, and 36 months.^28^ Following all MRI assessments, T1 and FLAIR images were reviewed for incidental findings of relevance (eg, cortical infarct, tumor, or normal pressure hydrocephalus), and participants with findings were notified within 1 week by telephone for a discussion. A primary care visit was encouraged within 21 days of any remarkable findings with the research study MRI provided to facilitate expedited care as deemed necessary. Adverse events (AEs) were monitored in real time with weekly staff review using clinical judgment and reports to the local institutional review board and an external data and safety monitoring board. The data and safety monitoring board convened biannually for updates on the progress and the review of study data including any AE coded by organ system under Medical Dictionary for Regulatory Activities format. Participants who withdrew from the study received an early termination visit either in person or by telephone to document withdrawal explanation and abbreviated follow-up plan for off-study treatment.

### Statistical Analysis

Data analysis was performed from February 2020 to July 2022. Statistical power and sample size were informed by the longstanding Oregon Brain Aging Study (OBAS) cohort of participants aged 80 years and older with CDR less than or equal to 0.5 and WML greater than or equal to 6 cm^3^, which was prevalent in 80% of the OBAS cohort. Simulation analysis estimated that the current trial placebo group should accumulate a mean (SD) of 7 (5) cm^3^ of WML over 3 years. However, during recruitment efforts, the prevalence of WML greater than or equal to 6 cm^3^ was 46.7% (109 of 233 participants), and documented protocol modifications were made to meet enrollment without sacrificing the primary hypotheses. Therefore, the threshold for WML inclusion was lowered to 5 cm^3^, age to 75 years or older, and sample size from 150 to 100 participants. Together, these modifications sustained 80% power to test the hypothesis that ω-3 attenuates annual WML progression by 50% compared with the placebo (ie, mean [SD], 3.5 [5.0] cm^3^ over 3 years in ω-3 group vs 7.0 [5.0] cm^3^ in the placebo group). Considering 30% attrition over 3 years, a total of 94 individuals were required, with 33 completers per treatment group.

Differences in annual change among study outcomes by treatment group were assessed using linear mixed-effects models, as described elsewhere,^28^ using R statistical software version 4.2 (R Project for Statistical Computing),^37^ with lme4,^38^ nlme,^39^ and ggplot2^40^ plot packages.^28^ Treatment stratification by *APOE* genotype was prespecified. Interaction terms between treatment and time evaluated annualized change differences by group. Model covariates included baseline age, sex, history of vascular disease (yes/no, indicating any one of the following: history of cardiac arrest, angina pectoris, angioplasty, cardiac bypass surgery, congestive heart failure, and atrial fibrillation), history of hypertension (yes/no), history of depression (yes/no), and MRI system used. MRI system was included as a covariate for the volumetric and diffusion MRI outcomes to account for system upgrade during the trial and MRI volumes (eg, WML, total brain volume, medial temporal lobe volume, and ventricular volume) were adjusted for intracranial volume at each MRI visit. Estimates and 95% CIs for WML were calculated on the original scale of cubic centimeters using linear mixed-effects models (ie, using pretransformed WML as outcomes) to provide crude annual volume change and same covariate corrections. Adverse events were analyzed using Fisher exact test in all randomized participants who received treatment. All hypothesis testing was 2-sided with results considered statistically significant at *P* < .05. Model fitness and confounding evaluations are provided in the eAppendix in Supplement 2.

## Results

### Enrollment, Demographics, and Attrition

**Figure 1.**
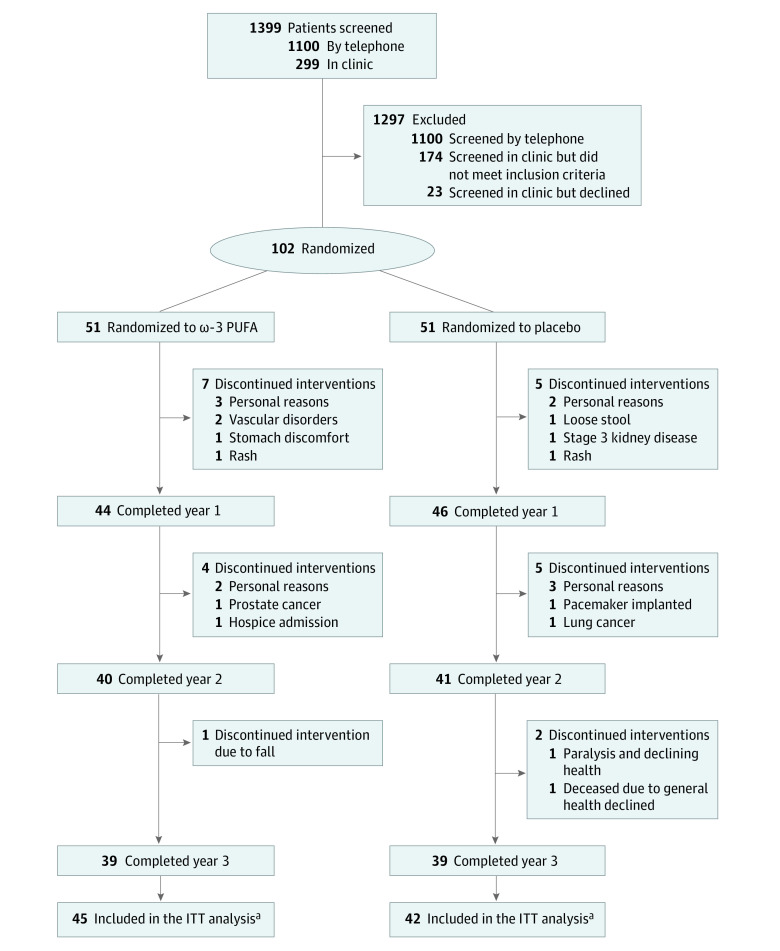
Participant Enrollment Flowchart PUFA indicates polyunsaturated fatty acid. ^a^The intention-to-treat (ITT) analysis includes all randomly assigned participants with at least 1 follow-up magnetic resonance imaging scan (n = 87).

Recruitment was initiated in May of 2014 and concluded in July 2016. The participants’ final visit was in September 2019. Figure 1 presents the participant enrollment and flow through the trial beginning with mass mailers and telephone screens, yielding 299 potential candidates for in-clinic screenings, of whom 102 individuals (62 women [60.8%]; mean age, 81 years [range, 75-96 years]) met inclusion criteria for both WML greater than or equal to 5 cm^3^ and plasma ω-3 PUFA less than 11.0 mg/dL or less than 5.5% whole-blood spot and agreed to be randomized (1:1; 51 per group). Twenty-eight participants (28%) carried an APOE*E4 genetic allele. The mean (SD) MMSE score was 28.0 (1.7), and the mean (SD) score MoCA was 24.0 (3.2), with all participants considered to not have dementia (eg, MMSE >23 and CDR of ≤0.5) (Table 1). Ninety participants completed the 1-year visit (44 in the ω-3 group and 46 in the placebo group), 81 completed the 2-year visit (40 in the ω-3 group and 41 in the placebo), and 78 completed the final 3-year visit (39 per group). Forty-five participants in the ω-3 PUFA group and 42 in the placebo group had at least 1 follow-up MRI to calculate change by treatment group under the intention-to-treat (ITT) protocol. The annual rate of participant attrition over the 3-year duration was similar among the treatment groups (8.5%; *P* for difference = .32, Fisher exact test). As expected, much of the attrition occurred earlier in the trial (11.7% by the end of year 1, 20.5% by the end of year 2, and 23.5% by the end of year 3).

**Table 1.** Baseline Demographic and Clinical Characteristics^a^

Characteristic	Participants, No. (%) (N = 102)	
	ω-3 (n = 51)	Placebo (n = 51)
Age, mean (SD) [range], y	81.2 (4.4) [75-96]	81.1 (4.4) [75-93]
Sex		
Female	31 (60.8)	31 (60.8)
Male	20 (39.2)	20 (39.2)
Race and ethnicity		
African American or Black	1 (2.0)	0
Asian	3 (5.9)	0
White, Hispanic	3 (5.9)	1 (2.0)
White, Non-Hispanic	44 (86.3)	50 (98.0)
Education		
High school graduate or less	14 (27.5)	10 (19.6)
Some college	9 (17.6)	11 (21.6)
College graduate or advanced degree	28 (54.9)	30 (58.8)
Systolic blood pressure, mean (SD), mm Hg	146.7 (13.7)	145.2 (18.6)
Diastolic blood pressure, mean (SD), mm Hg	68.9 (10.7)	70.7 (13.0)
Body mass index, mean (SD)^b^	27.4 (4.8)	26.2 (3.8)
Apolipoprotien E ε4 carrier	13 (25.5)	15 (29.4)
Plasma ω-3 polyunsaturated fatty acid (20:5 + 22:6), mean (SD), mg/dL	7.135 (2.448)	6.662 (2.176)
Mini-Mental State Exam score, mean (SD)	27.6 (1.7)	28.2 (1.8)
Montreal Cognitive Assessment score, mean (SD)	24.0 (3.2)	24.7 (3.0)
Clinical Dementia Rating of 0	35 (68.6)	36 (70.6)
Instrumental Activities of Daily Living, mean (SD), No.	0.1 (0.5)	0.1 (0.2)
Geriatric Depression Scale score, mean (SD)	1.6 (1.6)	1.3 (1.3)
History of depression	11 (21.6)	12 (23.5)
History of hypertension	39 (76.4)	35 (68.6)
History of vascular disease	8 (15.7)	7 (13.7)

SI conversion factor: To convert polyunsaturated fatty acid to millimoles per liter, multiply by 0.0355.

^a^ Baseline covariates were evaluated and confirmed for balance across the 2 groups using t-statistics for continuous variables and χ^2^ statistics for categorical variables (sex, race/ethnicity, education, apolipoprotein E ε4 status, Clinical Dementia Rating of 0, histories).

^b^ Body mass index is calculated as weight in kilograms divided by height in meters squared.

### ω-3 PUFA Effects on Total WML Progression

**Figure 2.**
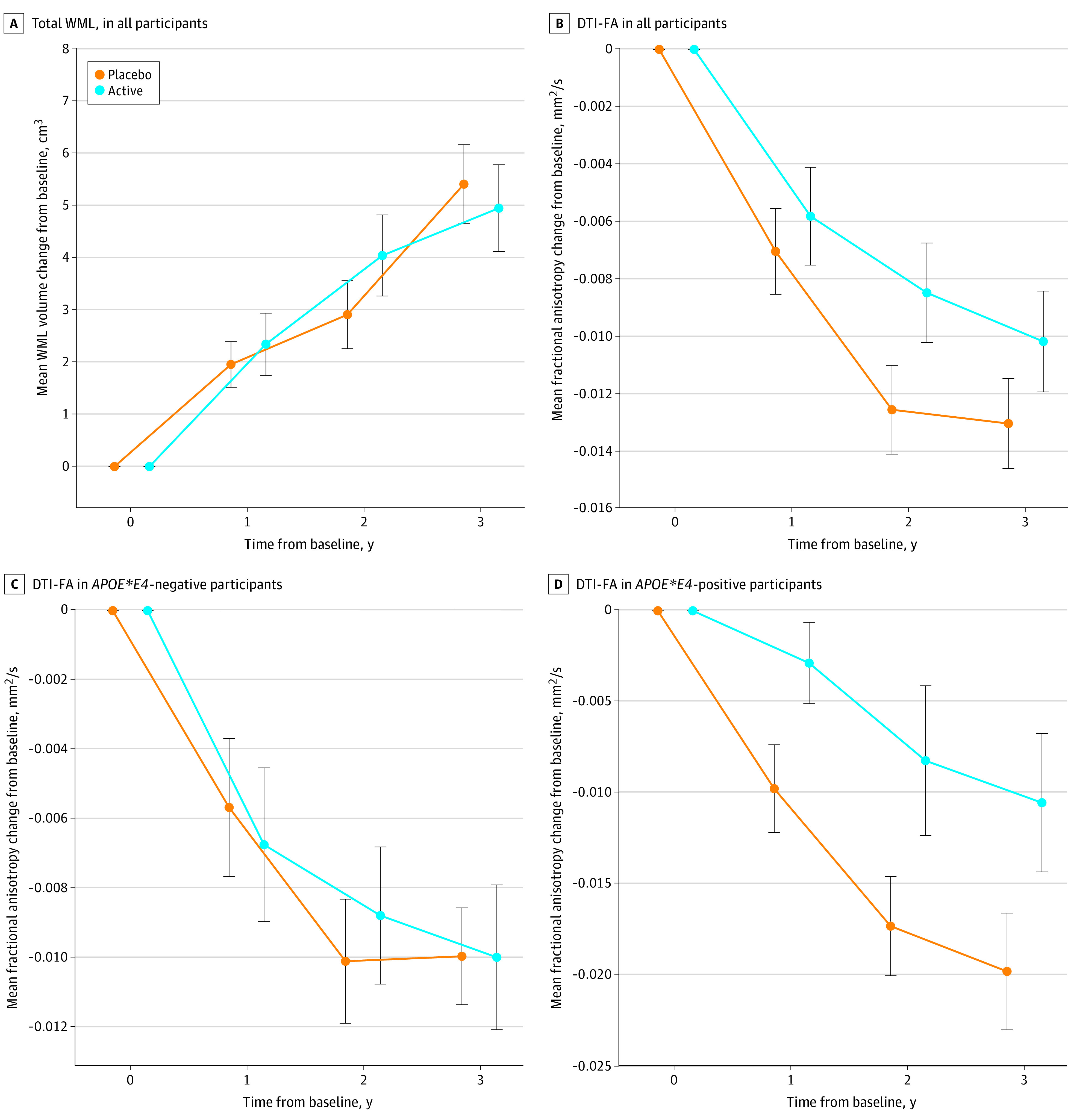
Effects of ω-3 Polyunsaturated Fatty Acids on White Matter Lesion (WML) and Neuronal Integrity Breakdown in All Participants and by Apolipoprotein E Genotype The intention-to-treat protocol includes all 87 randomized participants with at least 1 follow-up magnetic resonance imaging (MRI) scan. All linear mixed-effects models were adjusted for age, sex, hypertension, and other vascular disease, depression, MRI machine (and intracranial volume for WML). WML volume was back-calculated to crude values after modeling on log transformation. A, *P* = .30; B, *P* = .07; C, *P* = .66; and D, *P* = .04. Error bars denote SEM. DTI-FA indicates diffusion tensor imaging of fractional anisotropy.

Although the ω-3 group had less annual crude WML progression than the placebo group, the difference was not statistically significant (1.19 cm^3^ [95% CI, 0.64-1.74 cm^3^] vs 1.34 cm^3^ [95% CI, 0.80-1.88 cm^3^]; *P* = .30). The mean annual log-transformed WML progression between treatment groups also did not differ (0.08 cm^3^ [95% CI, 0.04-0.12 cm^3^] in the ω-3 group vs 0.10 cm^3^ [95% CI, 0.06-0.14 cm^3^] in the placebo). Table 2 shows log-transformed values, and Figure 2A shows the crude value changes.

**Table 2.** ω-3 Polyunsaturated Fatty Acid Effects on the Main Outcomes in All Participants and by APOE*E4 Carrier Status Over 3 Years Under the Intention to Treat Protocol^a^

Outcome	Annual change (95% CI)^b^		Linear difference between groups, coefficient estimate (95% CI)^c^	ω-3 Effect size estimates	
	ω-3 Group	Placebo group		*t* Statistic (*P* value)^d^	Cohen *d*^e^
Primary outcome					
White matter lesion, total, cm^3^ (log)	0.0782 (0.0414 to 0.1151)	1.0000 (0.0638 to 0.1357)	−0.0216 (−0.0624 to 0.0191)	−1.04 (.30)	−0.24
APOE*E4 positive	0.0417 (−0.0362 to 0.1197)	0.1067 (0.0418 to 0.1702)	−0.0650 (−0.1522 to 0.0195)	−1.50 (.15)	−0.63
APOE*E4 negative	0.0917 (0.0497 to 0.1337)	0.0980 (0.0543 to 0.1410)	−0.0064 (−0.0515 to 0.0394)	−0.27 (.79)	−0.07
Secondary outcomes					
Diffusion tenor imaging of fractional anisotropy, mm^2^/s	−0.0014 (−0.0027 to 0.0002)	−0.0027 (−0.0041 to −0.0014)	0.0014 (−0.0001 to 0.0028)	1.84 (.07)	0.42
APOE*E4 positive	−0.0015 (−0.0041 to 0.0011)	−0.0047 (−0.0067 to −0.0025)	0.0032 (0.0004 to 0.0062)	2.22 (.04)	0.92
APOE*E4 negative	−0.0012 (−0.0028 to 0.0004)	−0.0016 (−0.0032 to 0.0020)	0.0004 (−0.0013 to 0.0020)	0.44 (.66)	0.08
Medial temporal, cm^3^	−0.3664 (−0.4832 to −0.2496)	−0.3585 (−0.4731 to −0.2434)	−0.0079 (−0.1289 to 0.1134)	−0.13 (.90)	−0.03
APOE*E4 positive	−0.5656 (−0.7850 to −0.3462)	−0.4120 (−0.5922 to −0.2295)	−0.1536 (−0.3807 to 0.07412)	−1.31 (.20)	−0.56
APOE*E4 negative	−0.3129 (−0.4540 to −0.1718)	−0.3306 (−0.4758 to −0.1862)	0.0178 (−0.1236 to 0.1605)	0.24 (.81)	0.04
Whole brain, cm^3^	0.5352 (−3.6250 to 4.6960)	−1.8930 (−6.0280 to 2.1300)	2.4280 (−2.2360 to 7.2250)	1.01 (.32)	0.23
APOE*E4 positive	−3.0560 (−12.2300 to 6.1200)	−2.9360 (−10.7800 to 4.7530)	−0.1193 (−9.2680 to 9.2560)	−0.03 (.98)	−0.01
APOE*E4 negative	1.2940 (−3.2710 to 5.8580)	−1.3850 (−6.1210 to 3.1710)	2.6790 (−2.7500 to 8.2280)	0.96 (0.34)	0.26
Ventricular, cm^3^ (log)	0.0323 (0.0245 to 0.0401)	0.0343 (0.0267 to 0.0418)	−0.0019 (−0.0113 to 0.0076)	−0.40 (.69)	−0.09
APOE*E4 positive	0.0348 (0.0194 to 0.0502)	0.0334 (0.0206 to 0.0460)	0.00138 (−0.0152 to 0.0182)	0.16 (.87)	0.07
APOE*E4 negative	0.0325 (0.0230 to 0.0421)	0.0349 (0.0252 to 0.0446)	−0.00242 (−0.0144 to 0.0097)	−0.39 (.70)	−0.11

Abbreviation: APOE*E4, apolipoprotein E ε4 allele.

^a^ The intention-to-treat cohort includes all randomly assigned participants with at least 1 follow-up magnetic resonance imaging scan (n = 87).

^b^ Annualized changes were calculated from primary mixed-effects models as time trajectories for ω-3 and placebo groups.

^c^ Linear response difference was taken as the treatment-time interaction coefficient from the primary mixed-effects models.

^d^ Omega-3 treatment effect sizes were taken as the *t*-statistic from the primary mixed-effects model for the treatment-time interaction.

^e^ Cohen *d* was calculated from the primary mixed-effects models using *t*-statistic and appropriate *df* for each outcome.

### ω-3 PUFA Effects on Neuronal Integrity Breakdown and Brain Atrophy

Although the ω-3 group had less DTI-FA decline than the placebo group, the difference was not statistically significant (−0.0014 mm^2^/s [95% CI, −0.0027 to 0.0002 mm^2^/s] in the ω-3 group vs −0.0027 mm^2^/s in the placebo [95% CI, −0.0041 to −0.0014 mm^2^/s]; *P* = .07) (Table 2 and Figure 2B). Mean annual medial temporal lobe atrophy between treatment groups did not differ (−0.37 cm^3^ in the ω-3 group [95% CI, −0.48 to 0.25] vs −0.36 cm^3^ [95% CI, −0.47 to 0.24 cm^3^] in the placebo group; *P* = .90) (Table 2). The mean annual total brain volume change and ventricular volume change did not differ between the groups (Table 2).

### ω-3 PUFA Effects on Primary and Secondary Outcomes by *APOE* Genotype Under ITT

The mean annual increase in WMLs in APOE*E4 carriers treated with ω-3 vs APOE*E4 carriers treated with placebo was lower but did not reach statistical significance (0.04 cm^3^ [95% CI, −0.04 to 0.12 cm^3^] in the ω-3 group vs 0.11 cm^3^ [95% CI, 0.04 to 0.17 cm^3^] in the placebo group; *P* = .15). The mean annual increase in WML in APOE*E4 noncarriers treated with ω-3 vs APOE*E4 noncarriers treated with placebo did not differ (0.09 cm^3^ [95% CI, 0.05 to 0.13 cm^3^] in the ω-3 group vs 0.10 cm^3^ [95% CI, 0.05 to 0.14 cm^3^] in the placebo group; *P* = .79). The mean annual DTI-FA decline in APOE*E4 carriers treated with ω-3 was reduced compared with APOE*E4 carriers treated with placebo (−0.0016 mm^2^/s [95% CI, −0.0032 to 0.0020 mm^2^/s] in the ω-3 group vs −0.0047 mm^2^/s [95% CI, −0.0067 to −0.0025 mm^2^/s] in the placebo group; *P* = .04). Noncarriers of APOE*E4 treated with ω-3 vs noncarriers of APOE*E4 treated with placebo had similar mean annual DTI-FA decrease (0.0012 mm^2^/s [95% CI, 0.0028 to 0.0004 mm^2^/s] in the ω-3 group vs 0.0016 mm^2^/s [95% CI, 0.0032 to 0.0020 mm^2^/s] in the placebo group; *P* = .66) (Figure 2C and Figure 2D).

### Safety and Tolerability

Serious AEs (SAEs) or AEs between groups did not differ (Table 3). SAEs occurred in 16 participants (31.4%) across both groups (hospitalizations, *P* = .82; deaths, *P* = .36; Fisher exact test for difference between groups). Hospitalization prevalence was 25.4% (26 participants) over the course of the study (12 participants in the ω-3 group and 14 participants in the placebo group). Five deaths were recorded, including 4 in the ω-3 group: 1 due to a fatal fall, 1 renal and urinary tract disorder, 1 hematologic vascular disorder, and 1 prostate cancer metastasis. The 1 death in the placebo group was due to a nonhematological vascular disorder. No SAEs were deemed as attributable to the treatments. AEs occurred in 44 participants in the ω-3 group and 41 in the placebo group (*P* for difference = .78, Fisher exact test). The most common AEs were injurious falls (30.3%; 17 in the ω-3 group and 14 in the placebo group), musculoskeletal and connective tissue disorders (25.4%, predominantly preexisting joint conditions, such as osteoarthritis), and gastrointestinal disorders (24.5%; 10 participants in the ω-3 group and 15 participants in the placebo group; predominantly stomach pain, loose stool, gas, or flatulence). Twelve participants in the ω-3 and placebo groups discontinued study treatment due mainly to AEs classified as unrelated to the experimental treatments. There was no dropout difference due to SAEs or AEs among the groups (7 participants). The dropout rates due to AEs were 11.7% (6 participants) in the ω-3 group and 5.9% in the placebo group (3 participants) (*P* for difference = .49, Fisher exact test).

**Table 3.** Adverse Events by Experimental Group

Event^a^	Participants, No. (%)	
	ω-3 (n = 51)	Placebo (n = 51)
At least 1 serious adverse event	16 (31.4)	16 (31.4)
At least 1 adverse event	44 (86.3)	41 (80.4)
Serious adverse events		
Hospitalizations^b^	12 (23.5)	14 (27.4)
Vascular disorders nonhematological	0	3 (5.8)
Renal and urinary disorders	2 (3.9)	2 (3.9)
Cardiac disorders	2 (3.9)	2 (3.9)
Gastrointestinal disorders	1 (1.9)	0
Injury, poisoning, and procedural complications	4 (7.8)	0
Deaths^c^	4 (7.8)	1 (1.9)
Injury, poisoning, and procedural complications	1 (1.9)	0
Vascular disorders, nonhematological	0	1 (1.9)
Renal and urinary disorders	1 (1.9)	0
Vascular disorders, hematological	1 (1.9)	0
Neoplasms benign, malignant, and unspecified	1 (1.9)	0
Adverse events		
Injury, poisoning, and procedural complications	17 (33.3)	14 (27.4)
Musculoskeletal and connective tissue disorders	12 (23.5)	14 (27.4)
Gastrointestinal disorders	10 (19.6)	15 (29.4)
Nervous system disorders	12 (23.5)	13 (25.4)
Respiratory, thoracic, and mediastinal disorders	11 (21.5)	10 (19.6)
Psychiatric disorders	9 (17.6)	8 (15.6)
Vascular disorders, hematological	9 (17.6)	4 (7.8)
Neoplasms benign, malignant, and unspecified	5 (9.8)	8 (15.6)
General disorders and administration site conditions	6 (11.7)	4 (7.8)
Cardiac disorders	3 (5.8)	5 (9.8)
Renal and urinary disorders	4 (7.8)	4 (7.8)
Infections and infestations	5 (9.8)	2 (3.9)
Surgical and medical procedures	3 (5.8)	4 (7.8)
Vascular disorders nonhematological	1 (1.9)	5 (9.8)

^a^ Adverse events are defined according to the Medical Dictionary for Regulatory Activities terminology. Rates between groups across categories were similar (Fisher exact test, *P* = .60).

^b^ Fisher exact test, *P* = .82.

^c^ Fisher exact test, *P* = .36.

### ω-3 PUFA Effects on Exploratory Outcomes Regional WML, DTI-Radial, Mean, and Axial Diffusivity, and Executive Cognitive *z*-Score

The mean annual periventricular WML increase was 0.09 cm^3^ (95% CI, 0.05 to 0.13 cm^3^) in the ω-3 group and 0.11 cm^3^ (95% CI, 0.07 to 0.15 cm^3^) in the placebo group (*P* = .28). The mean annual increase in subcortical WML was 0.03 cm^3 ^(95% CI, −0.04 to 0.09 cm^3^) in the ω-3 group and 0.04 cm^3 ^(95% CI, −0.02 to 0.11 cm^3^) in the placebo group (*P* = .72). Mean annual DTI radial diffusivity increase was 3.143 × 10^−6^ mm^2^/s (95% CI, 1.449 × 10^−6^ to 4.836 × 10^−6^ mm^2^/s) in the ω-3 group and 4.894 × 10^−6^ mm^2^/s (95% CI, 3.280 × 10^−6^ to 6.500 × 10^−6^ mm^2^/s) in the placebo group (*P* = .06), and the mean annual DTI mean diffusivity increase was 2.882 × 10^−6^ mm^2^/s (95% CI, 1.377 × 10^−6^ to 4.386 × 10^−6^ mm^2^/s) in the ω-3 group and 4.174 × 10^−6^ mm^2^/s (95% CI, 2.736 × 10^−6^ to 5.602 × 10^−6^ mm^2^/s) in the placebo group (*P* = .10). There were no differences between group in executive cognitive function *z* scores (eTable 3 in Supplement 2).

## Discussion

This randomized clinical trial in older adults with suboptimal ω-3 status and WMLs, the neurovascular pathology thought to be impacted by the intervention, examined whether ω-3 treatment can slow WML progression over 3 years. Although the treatment was safe and well tolerated, it failed to significantly reduce WML compared with the soybean oil placebo. However, some differential effects by *APOE* genotype were identified in a prespecified secondary outcome representing reduction in neuronal integrity breakdown with ω-3 intervention.

Our enrollment of participants aged 75 years and older with WML burden (≥5 cm^3^) and suboptimal ω-3 status (<11.0 mg/dL or 5.5 weight percentage) was successful in that there was an accelerated rate of WML accumulation compared with previous studies.^41,42^ However, preliminary data from the OBAS had a mean WML accumulation of 7 cm^3^ over 3 years, whereas a total of 4 to 5 cm^3^ of WML accumulation was observed in the current trial.^43^ Less WML accumulation would dilute the statistical power and increase the number of individuals needed to see change and possibly affect the duration of treatment needed to observe efficacy. This may explain why the ITT analysis failed to reach statistical significance for the primary outcome of annual WML, secondary outcome of DTI-FA, and exploratory outcomes of DTI-radial diffusivity and mean diffusivity in the ω-3–treated group. Although ω-3 effects on WML progression in APOE*E4 carriers did not reach significance, APOE*E4 carriers treated with ω-3 had significantly less neuronal integrity breakdown (DTI-FA). The larger neuronal integrity breakdown seen in APOE*E4 carriers that we (and others^44,45^) have observed further supports sample size as a potential important limitation to our ability to detect universal effects of ω-3 on WML and DTI-FA among all participants.^46^ This trial was not powered to detect differences between APOE*E4 carriers and noncarriers and may contribute to the nonsignificant direct comparisons. Regardless, the differential effects of ω-3 we observed on neuronal integrity breakdown by *APOE* genotype has yet to be reported and requires further confirmation.^47^

The ω-3 PUFA formula using both EPA and DHA combined but more concentrated with EPA was justified because both are brain penetrant lipids,^48^ plasma EPA plus DHA explains 24% of the total WML volume in older adults, and EPA had the largest association with less WML (ie, largest loading coefficient from the in silico model).^19^ EPA is also more abundant than DHA in the microglia and has a more profound ability to reduce microglial proinflammatory cytokine release and endothelial cell activation that permits peripheral immune cell entry to the brain.^24,49,50,51^ Clinical trials of 18 months and longer that have tested ω-3 at 1 g or more have either administered DHA solely or a DHA-dominant formula with small quantities of EPA. For example, the Multi-domain Alzheimer Prevention Trial randomized people to 3 years of a lifestyle intervention with and without DHA of 800 mg and EPA of 225 mg but failed to achieve cognitive benefit.^52^ Another trial used DHA of 2 g daily for 18 months for individuals with mild-to-moderate AD and failed to achieve effects on AD progression and neuroimaging outcomes of whole brain and hippocampal volume atrophy.^30^ However, DHA did reduce Alzheimer Disease Assessment Scale–Cognitive progression in noncarriers of the APOE*E4 allele.^30^ The current trial of an EPA-dominant formula failed to significantly reduce WML progression in all participants, but neuronal integrity breakdown in APOE*E4 carriers did appreciate benefit as early as 1 year after ω-3 treatment. Together these findings suggest that an EPA-dominant formula may provide some benefit in APOE*E4 carriers with no dementia and WMLs, and DHA-dominant formulas may benefit noncarriers of APOE*E4 with mild-to-moderate AD. Trial formula daily doses of DHA range from 0.650 to 2 g and from 0 to 0.975 g of EPA, and the treatment duration range is from 6 months to 3 years. The disease stage of each interventional trial has also varied from older, community-based, cognitively intact individuals to those with mild cognitive impairment to those with mild-to-moderate AD. All these trials thus far could be augmented with adherence biomarkers to ensure that the hypothesized neuroprotective blood thresholds observed in preliminary studies are achieved by each participant by trial study exit.^27,28^ As a result of substantial heterogeneity in ω-3 dosages and formula and the duration of treatment and disease stage of participants, more targeted interventions are needed that build on knowledge gained from these past well-executed trials before drawing firm conclusions for the role that ω-3 may play in dementia prevention.

Strengths of the current trial include a unique design leveraging blood and MRI biomarkers to target individuals with the evidence of the neuropathologic entity thought to benefit from the intervention, and exclusion of people already within the hypothesized neuroprotective threshold for plasma ω-3.^53^ The annual neuroimaging measures as surrogate indicators of future cognitive impairment to demonstrate ω-3 target engagement, and in a population of older adults often underrepresented in dementia-focused trials, is also an asset to the field.^54^ The independent laboratory testing of the natural product ensured the purity and potency over the course of study. The systematic biospecimen banking to permit per-protocol analyses in the future comparing participants who achieved the prespecified neuroprotective plasma threshold of ω-3 to those who did not reach that threshold over the trial course (ie, 11.0 mg/dL EPA plus DHA). We believe these design elements are fundamental to draw firm conclusions from clinical nutrition trials.

### Limitations

This study has limitations that should be mentioned. The population was demographically and geographically homogeneous, which may limit the generalizability of the findings. A future multisite trial will have the ability to enroll a more ethnically, racially, and geographically diverse population and provide adequate sample size to permit assessment of clinical benefit.^55^ The Tim Trio scanner was upgraded to a Siemens 3-T Prisma during the study; however, all baseline MRI scans were performed before the upgrade and the analytical approach was adjusted for MRI system as a time-varying covariate when evaluating imaging outcomes. As further validation, assessment of participants who completed the study before and after the upgrade had no differences in WML and DTI-FA as a function of the MRI system. The ω-3 effects on whole-brain DTI-FA includes WML across voxels; therefore, the effects on DTI-FA independent of WMLs in APOE*E4 carriers remains an open question. WML progression was less than anticipated, and, therefore, this trial may have been underpowered to reasonably detect WML effects under ITT. Similarly, this study was not powered to directly compare effects between APOE*E4 carriers and noncarriers.

## Conclusions

In this study, ω-3 treatment was safe and well-tolerated but failed to reach significant reduction in WML progression and neuronal integrity breakdown among all participants at risk for dementia. However, the significant reduction in neuronal integrity breakdown among APOE*E4 carriers suggests that the effects of ω-3 may be amplified for these individuals. These results will enable improved study design and sample size calculations for future efforts of a relatively cheap, safe, and well-tolerated therapy for primary and secondary dementia prevention.

## Notes

Supplement 1.
Trial Protocol and Statistical Analysis Plan

Supplement 2. **eTable 1.** PUFA trial eligibility criteria **eTable 2.** Independent laboratory testing of ω-3 PUFA and soybean oil placebo illustrating a potent (total mg/mL oil/capsule and % concentration of label claim) and stabile (lipid oxidation^a, b, c^) supplement over trial duration **eAppendix.** Supplemental methods **eTable 3.** ω-3 PUFA effects on exploratory outcomes and by apoE genotype over 3-years^a^
eReferences

Supplement 3.
Data Sharing Statement
