# Supplementary material for: ω-3 PUFA for Secondary Prevention of White Matter Lesions and Neuronal Integrity Breakdown in Older Adults: A Randomized Clinical Trial
Source: JAMA Netw Open. 2024 Aug 1;7(8):e2426872. doi: 10.1001/jamanetworkopen.2024.26872 (PMC11294966; doi:10.1001/jamanetworkopen.2024.26872)
Supplement: Supplement 1. — Trial Protocol and Statistical Analysis Plan [file jamanetwopen-e2426872-s001.pdf]

Study Protocol
Principal Investigator: Drs. Gene Bowman and Lynne Shinto
IRB#: 9880
Study/Protocol Title: Omega 3 pufas for the vascular component of age-related cognitive decline

SPECIFIC AIMS

An abundance of epidemiological evidence exists in favor of nutritional factors for reducing dementia risk. Yet trials formally testing nutritional therapies have been disappointing. We now have evidence showing that nutrients act through distinct pathways that contribute to dementia risk, some acting more through vascular pathology mediators while others may influence brain pathology more directly. This phenomenon could explain why well-executed nutritional interventions for AD have been inconclusive and suggests that subsequent clinical trials require a new approach. There is good reason to believe that the effects of omega 3 polyunsaturated fatty acids (pufas) on brain function predominantly operate through vascular mechanisms. We provide new results that indicate a relationship between plasma pufas, total cerebral white matter hyperintensity (WMH) volume and cognitive decline in domains sensitive to brain white matter disease, with higher plasma levels of omega 3 pufas associated with less white matter disease and less cognitive decline. These results raise the question of whether the omega 3 pufas can favorably modify vascular contributions to cognitive decline, resulting in improved endothelial function, reduced accumulation of WMH and ultimately more stable cognitive function. Since WMH appears to be a reliable and valid biomarker of cerebral small vessel disease, and a risk factor for cognitive decline, we propose a trial which uses this objective MRI-derived measure as the primary outcome. The primary aims include:

Aim 1: Determine if omega 3 pufa supplementation slows the accumulation of total white matter hyperintensity volume (WMH) in non-demented elders. We (and others) have demonstrated that total WMH volume and accumulation can accelerate cognitive decline in non-demented elders. Pufas may affect dementia risk when a major vascular component is present, namely WMH. This aim will test the primary hypothesis that pufas slow the total WMH accumulation in persons of advanced age (≥75) compared to placebo. This aim will also examine pufa effects on medial temporal lobe atrophy and regional fractional anisotropy in white matter tracts within the inferior frontal gyri using diffusion tensor imaging.

Aim 2: Determine if omega 3 pufa supplementation improves blood-based biomarkers of endothelial function. Soluble adhesion molecules are elevated in atherosclerosis, stroke and cardiovascular disease representing endothelial dysfunction. The incorporation of omega 3 pufas into the vascular endothelium alters cell structure and metabolism. This aim will test the hypothesis that pufas lower biomarkers of endothelial dysfunction, plasma soluble adhesion molecules (e.g. ICAM-1). This aim will also test pufa effects on plasma biomarkers of other inflammatory molecules, and on amyloid and lipid metabolism.

Aim 3: Determine if omega 3 pufas slow decline in psychometric indices historically sensitive to WMH accumulation. We (and others) have demonstrated plasma or intake of pufas associated with cognitive function and decline, and dementia incidence, but formal trials in AD have not shown efficacy. New evidence indicates that, in contrast, the pufa effects on cognition are best appreciated in domains sensitive to WMH accumulation, executive function and processing speed. This exploratory aim tests the hypothesis that pufas preserve executive function and processing speed in non-demented persons of advanced age (≥75) with WMH at baseline over 3 years.

Targeting the vascular component of age-related cognitive decline with emphasis on total WMH and endothelial function is a novel approach. We have experience and access to a unique study cohort, a population enriched by advanced age and vascular risk. The excellent safety profile of our proposed intervention is notable. Our research team has expertise in pufa supplementation and metabolism, clinical and trial experience with pufas, neuroimaging and neuropsychological evaluation in persons of advanced age.

Hypothetical model for pufa effects on age-related cognitive decline

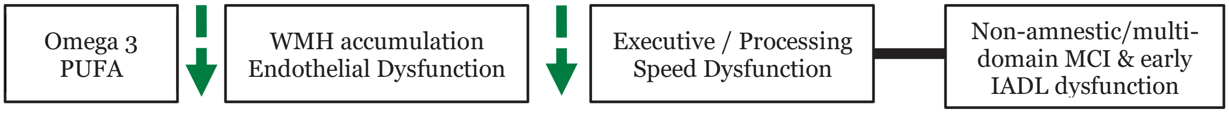

SIGNIFICANCE

A1. The vascular components of age-related cognitive decline are significant, but are not being targeted in dementia prevention trials. Several recent reviews have examined studies that support the significance of vascular disease in the pathogenesis of all-cause dementia[3, 4], and have concluded that reduction in vascular risk could dramatically reduce the prevalence of all-cause dementia[5, 6]. While Alzheimer's disease prevention efforts continue to focus on "anti-amyloid" approaches, reducing vascular risk may ultimately prove most effective for reducing age-related cognitive decline.

A2. The paradigm in dementia prevention studies needs to incorporate reproducible and valid measures of the vascular components significant to the brain. This will help facilitate the testing of experimental interventions more efficiently. Dementia prevention studies are very challenging in the general population, requiring enrollment of thousands of subjects for studies lasting 5 or more years[7]. To reduce the potential number of subjects needed and the duration of study, validation of appropriate surrogate markers of disease is critical. Cerebral white matter hyperintensity (WMH) volume is an important vascular marker related to age-related cognitive decline and the risk for all-cause dementia[3, 8, 9], and appears sensitive to vascular interventions[10]. However, this MRI-derived biomarker has not been evaluated as a primary outcome measure to our knowledge. The proposed study will provide an initial evaluation of WMH as a surrogate biomarker of disease risk in a non-demented cohort of advanced age.

Figure 1. Higher intake (A) and plasma (B) pufas are consistently associated with less brain white matter disease in non-demented elders

A

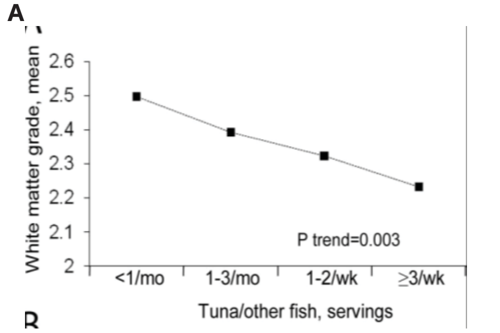

A: The graph above demonstrates the incremental decline in WMH grade by level of increasing fish consumption (adjusted for age, sex, race, enrollment center, diabetes, education, smoking, pack-years of smoking, BMI, coronary heart disease, ETOH use, physical activity, energy intake, meat consumption, and vegetable consumption. Adapted from Cardiovascular Health Study[1]

B

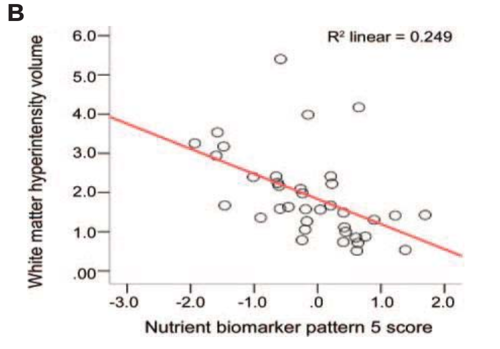

B: Our scatter plot showing a consistency with the Cardiovascular Health Study of less WMH disease with higher plasma pufas (nutrient biomarker pattern 5 is a standardized score representing plasma omega 3 pufas, EPA and DHA)(WMH volume reported as a % of total supratentorial brain volume)(adjusted for age, gender, education, APOE4 genotype and hypertension, p=0.03, n=42). Adapted from the Oregon Brain Aging Study[2]

A3. Emphasis on WMH as a vascular component is of significant interest to dementia prevention strategies. The prevalence of cerebral WMH in non-demented elders age 65 and older is estimated at 60 - 92%[11, 12]. Cross-sectional studies have shown WMH associated with worse cognitive function, worse scores on tests of activities of daily living and a higher likelihood for dementia [13-18]. Prospective observational studies have found baseline WMH volume as a strong predictor of the progression of WMH[19-22]. Our group[8, 9] and others have also observed an increased risk for cognitive decline as WMH progress in non-demented elders [23-27]. Although the cognitive phenotype associated with WMH progression may eventually pervade several cognitive domains, the literature provides consistent evidence in support of executive dysfunction with impaired information processing and cognitive flexibility as early indicators of underlying WMH accumulation[14, 18, 28-30]. MRI biomarkers of cerebral WMH are readily available to expedite the testing of therapeutic strategies.

A4. Evidence for cognitive benefits of pufas are in danger of being disregarded on the basis of clinical trials targeting non-vascular mechanisms. Observational studies draw attention to an important link between fish intake or plasma omega 3 pufa levels and lower WMH volume. One group examined the prevalence (n=4128) and incidence (n=1124) of silent infarcts and differences in WMH grade by fish consumption in the Cardiovascular Health Study, which included people free of any cerebrovascular disease and age 65 and older[1]. After adjustment for important risk factors, higher fish consumption was associated with less WMH burden (**figure 1A**), but not associated with MRI measures of more whole brain atrophy (sulcal and ventricular grades). In another study, our group utilized nutrient biomarker pattern analysis to circumvent the potential for recall bias when asking older people to recall their diet while simultaneously examining the nutrition most readily assessable by the brain.[31] This cross-sectional analysis examined the association of nutrient biomarker patterns with cognitive function (n=104, mean age 87, 62% women, 10% ApoE4 carriers) and MRI measures of WMH and total cerebral brain volume in a subset (n=42, mean age of 92, range 85-101). All study participants were non-demented.

After adjustment for important covariates, higher plasma levels of a nutrient biomarker pattern 5, distinctly representing the combination of eicosapentaenoic acid (EPA) and docosahexaenoic acid (DHA) was associated with less WMH volume (**figure 1B**). Interestingly, no relationship was detected between the plasma pufas and total cerebral brain volume; consistent with the Cardiovascular Health Study results[1]. After adjusting for age, gender, education, APOE4, hypertension and depression we identified the plasma pufas associated with better executive function and no relationship with global cognitive function, memory, attention, visuospatial skills or language (Trails B, $\beta = -0.24$, $p < 0.05$). The consistency and biological plausibility for these results are further supported by previous observation that WMH accumulation appears to disturb executive function predominantly[14, 18, 29, 30]. Altogether, these data suggest that pufa effects are more confined to the vascular pathology mediators of age-related cognitive decline (e.g. WMH) rather than the underlying mechanisms that govern total brain atrophy in late life (i.e., Alzheimer's type pathology). The Preliminary Study section presents the results of a new longitudinal study that further supports the hypothesis that pufa modulates "white matter mediated" cognitive decline (under review, *Annals of Neurology*).

Abundant additional evidence supports a role for pufas in the attenuation of vascular components of age-related cognitive decline. Placebo-controlled randomized trials of traditional vascular risk factors (e.g., blood pressure, cholesterol, diabetes) are unethical because placebo patients would be denied the standard of care. In contrast, a randomized trial using the omega 3 pufas is reasonable because this practice is currently outside the standard of care. Numerous observational studies have shown reduced risk of dementia in subjects consuming higher amounts of pufas, but these have never been directly tested in a randomized controlled dementia prevention trial. Yet there are several plausible explanations for pufas operating through vascular mechanisms in order to promote cognitive health: 1) Pufa consumption has well established effects on vascular health and is currently included in the standard of care for primary and secondary prevention of coronary heart disease [32]; 2) On the other hand, pufa trials in people with Alzheimer's disease, which select subjects with low vascular risk factors as a strategy for a pure AD experiment, have not seen efficacy[33, 34]; 3) Both observational studies and clinical trials have provided evidence that the beneficial effects of omega 3 pufas may be confined to non-carriers of the apolipoprotein E epsilon 4 allele[34, 35]. Since e4 carriers are more prone to cerebral amyloid than non-carriers, the observation that pufas may be more effective in non-carriers suggests that pufas operate via an amyloid-independent mechanism. This trial will collect plasma amyloid metabolites as a secondary measure to determine whether pufas are indeed independent from amyloid mechanisms[36-38] and we will covary for APOE4 status in secondary analysis to learn more.

PUFAs reduce inflammatory and atherogenic gene expression confirming their biological role in reducing inflammatory and atherogenic phenotypes. Many studies have demonstrated that omega 3 pufa (EPA+DHA) supplementation readily increases cell membrane concentrations, which alter the chemical (e.g. cell signaling) and structural (e.g. fluidity) properties of the cell. This is thought to be a key feature of their biological effects. In a study of patients awaiting carotid endarterectomy (n=121), volunteers were randomized to receive pufas or placebo over a median of 21 days prior to surgery. During the procedure tissue samples were collected and analyzed. The pufa group had significantly higher enrichment of EPA in the phospholipids of the carotid plaques ($p<0.0001$) and fewer foam cells ($p=0.03$). The pufas also attenuated gene expression (mRNA) for intercellular adhesion molecule 1 (ICAM-1, $p=0.01$), IL-6 ($p=0.03$), matrix metalloproteinases-7 ($p=0.005$), -9 ($p=0.004$) and -12 ($p=0.004$) further supporting the effects of pufas on inflammatory and cell integrity or stability[39]. Another trial in healthy elders demonstrated pufa supplementation of 1.8 g/d (EPA+DHA) over 26 weeks was capable of reducing gene expression involved in inflammatory and atherogenic-related pathways, including nuclear transcription factor kappa B signaling, eicosanoid synthesis and scavenger receptor activity[40].

PUFAs may have anti-leukocyte migration effects, which may be one explanation for their relationship with white matter mediated cognitive decline. One inflammatory protein and surrogate marker of endothelial function of particular interest is soluble ICAM-1, also known as CD54, a nearly ubiquitous transmembrane glycoprotein that plays a significant role in leukocyte migration and activation[41, 42]. The principal binding partners of ICAM-1 are the leukocyte integrin's LFA-1 (CD11a/CD18) and Mac-1 (CD11b/CD18)[43, 44]. At sites of inflammation, ICAM-1 is upregulated on endothelial and epithelial cells where it mediates the adhesion and paracellular migration of leukocytes expressing activated LFA-1[45]. ICAM-1 ligation prolongs antigen presentation by dendritic cells and promotes T cell proliferation and cytokine release[46].

Some evidence suggests that the pathogenesis of cerebral small vessel disease includes the expression of sICAM-1[47]. Soluble adhesion molecules are elevated in symptomatic ischemic stroke and also associated with WMH progression independently from other vascular risk factors[48, 49]. One observational study of 175 non-demented and stroke-free adults aged 60 and older found plasma sICAM-1 elevated in subjects with a higher Fazekas grade of WMH volume on the magnitude of 4.6 times higher odds of having white matter lesions in those subjects in the highest quartile of plasma sICAM-1[50]. However, in another study, higher plasma soluble adhesion molecules were elevated only in subjects with periventricular WMH and symptomatic carotid arterial stenosis and not different by WMH grade alone[51]. A recently published meta-analysis of 18 different RCTs also concluded that pufa supplementation attenuates plasma ICAM-1[52]. The Approach section presents preliminary studies in OBAS and ADNI non-demented elders showing plasma ICAM-1 association with cognitive decline and WMH progression, respectively.

A5. Why focus on individuals in advanced age (i.e. ≥ 75)? There are several important reasons to focus this study on the oldest old, and few strong arguments against their representation in clinical trials, including dementia prevention studies. This group is the fastest growing segment of the American population, is at very high risk of cognitive decline and dementia[53], and has a high prevalence of WMH[8, 9]. The neuropathology in subjects that succumb to dementia at this age is also less dominated by beta-amyloid plaques, tangles (compared to "young old" subjects) and APOE4 genotype[54, 55]. This supports our focus in the advanced age population for targeting a vascular mechanism. Despite the importance of this population in planning for future public health efforts to prevent late life dementia, these individuals are rarely included in clinical research.

Significance Summary. This proposal represents an opportunity to test a promising prevention strategy using a novel, biomarker-based approach, in a large and expanding population that is at high-risk and generally under-represented in these types of studies. Pufas have known effects on vascular health (lower triglycerides, blood pressure, coronary heart disease mortality and sudden cardiac related death), and some studies support a lower risk for ischemic stroke[56-58], recurrent ventricular arrhythmias [59] and congestive heart failure[60], all of which may modify risk for dementia. More recent brain research shows plasma or intake of pufas associated with less total WMH volume[1, 2], better executive function[2] and less executive decline (Approach Preliminary Studies). Pufa supplementation improves plasma biomarkers of endothelial function (ICAM)[52] and we show preliminary results of an association between plasma ICAM and WMH progression and cognitive decline (see Approach, Prelim Studies). In summary, pufas may improve endothelial function and slow WMH accumulation, which prevents white matter mediated cognitive decline appreciated earliest in executive function tasks

INNOVATION

B1. The study population is unique. Strategies to halt the progression of dementia have yet to show efficacy, and several explanations for this circumstance have been proposed. Patients with dementia have more rapid rates of decline; thus, they can provide more power to detect differences in a trial setting with a smaller sample size. However, in non-demented elders the slow rate of cognitive decline makes the design and feasibility of a dementia prevention trial challenging. The large Ginkgo trial [7] is an example of the challenges we face, where the projected dementia incidence was only 1% at completion of year one, far below the *a priori* estimate of 4% in persons 75 and older. This circumstance can deflate the ability to rigorously test efficacy, since the intention to treat analysis assumes high adherence to the study medication, and adherence drops dramatically and classically in trials of long duration leading to a regression to mean scenario. This trial is an example of the need for surrogate outcomes measures in the dementia prevention trial setting that can detect effects using smaller cohorts followed for shorter periods. Our study will enroll a cohort enriched with persons of advanced age and on the basis of their vascular risk (WMH volume) as complementary strategies. These attributes will provide a unique study population, "rich" in the characteristics that drive change in our outcomes of interest (age and WMH burden).

B2. The safety profile of the intervention is unique among dementia prevention strategies. Although the elderly have more rapid rates of change in brain structure and function, affording greater power to detect differences over shorter periods with fewer subjects, they also carry a greater likelihood for deleterious effects from study medications. The Women's Health Initiative Study [61] and the Alzheimer's Disease Anti-inflammatory Prevention Trial (ADAPT) [62] are both good examples of the adverse effects faced in the elderly using conjugated estrogens and NSAIDS, respectively. Both of these trials were aborted due to toxicity

experienced in the subjects and underscore the need for interventions with excellent safety profiles for wide use in older populations. The pufas are unique in this respect.

B3. Targeting the vascular component of cognitive decline and emphasizing WMH volume as the primary outcome measure is a novel approach. The proposed study will provide an initial evaluation of WMH as a surrogate biomarker of disease risk in an oldest old, non-demented cohort. This evaluation of WMH will occur independent of the intervention results and guarantee critical insight into its utility for future research.

B4. This proposal is distinct from previous prevention trials using pufas in terms of the target mechanism (vascular, endothelial function), primary outcomes (biomarker based MRI), and the population enrichment strategy (advanced age, low plasma pufa, and WMH burden). Briefly, the trial conducted by van de Rest et al[63] enrolled non-demented subjects age 65 and older and followed them for 26 weeks. Their population was younger and follow up was only 26 weeks. Dangour et al[64] enrolled a younger population range than the current proposal (age 70-79 vs ≥ 75) and this population was not enriched by any means of vascular risk, the pufa dose was half of that which we propose (700mg vs 1650) and measures of executive function and processing speed were not primary outcomes. Geleijnse et al[65] did include people with a history of vascular risk (CHD), but the cohort was younger (60-80 vs. ≥ 75), the pufa dose was 75% lower (400mg EPA+DHA vs. 1650), and the primary outcome was global (MMSE), not the executive and processing speed skills that appear most sensitive to pufas. In summary, our proposal takes advantage of new evidence that suggests pufas acts on vascular pathology mediated cognitive decline in non-demented elders. Our primary outcome and target mechanism (WMH and endothelial function), study population (advanced age, low pufa status and WMH burden) are evidence based rather than choosing outcome measures that are generically associated with age related cognitive decline. This makes the present proposal distinct from previous trials.

APPROACH

Preliminary Studies in the Oregon Brain Aging Study (PI: Jeffrey Kaye)**.** Clinical, neuro-psychometric and MRI measures have been collected annually since 1989 by a neurologist, neuropsychologist, and research staff. Beginning circa 2006-07 blood was collected for a sub-study of nutrient biomarkers (PI: Bowman K23) in order to examine the relationship of plasma pufas and endothelial function (ICAM) with brain function and structure in non-demented elders (CDR > 0.5 excluded). Baseline characteristics of the population are in Table 1. In the following results, all subjects are included in the analysis of pufas and cognitive decline, while only those with MRI are included in the WMH studies.

Table 1. Baseline characteristics of non-demented elders in OBAS with longitudinal data available for analysis

	***n*=86**
Age, y	85.7 (10)
Female, No./Total (%)	53/86 (62)
Years of Education	15 (3)
APOE4 carrier, No./total (%)	9/86 (11)
Hypertension, No./total (%)	36/85 (42)
Depression, No./total (%)	15/86 (17)
Clinical Dementia Rating of 0, No./Total (%)	60/86 (70)
Years of follow–up, mean (min/max)	4.0 (1/6)
**Plasma biomarkers**	
PUFA index (EPA+DHA), ug/mL	84.5 (26.7)
Eicosapentaenoic acid (EPA, 20:5n-3), ug/ml	16.5 (10.5)
Docosahexaenoic acid (DHA, 22:6n-3), ug/ml	68.1 (17.8)
ICAM-1, ng/ml	250.5 (74.3)
**Cognitive profile**	
MMSE	28 (2)
Trail Making Test Part B	137 (77)
Digit Symbol Making Test	39 (13)
Delayed paragraph recall[1]	14.0 (4.7)
**Brain MRI profile**	**n=32**
Total Intracranial Volume, cm^3	1107.5 (106.4)
Total Brain Volume, cm^3	821.1 (84.7)
Total Ventricular Volume, cm^3	55.2 (16.5)
White matter hyperintensity (WMH), cm^3	13.8 (8.8)

Mean and standard deviation unless otherwise stated
[1]WMS-R logical memory-delayed paragraph recall

***<u>Study 1</u>: Executive function is the cognitive domain that appears most sensitive to pufa modulation*.** After adjusting for age, gender, education, *APOE4*, hypertension, depression, and baseline PUFA index, higher plasma PUFA index (EPA+DHA) was associated with slower executive decline (Trails B)(Table 2). Each 100 ug/ml increase in the PUFA index was associated with 4 seconds less time to complete Trails B for each year increase in age (β = -0.04; 95% CI -0.07 to -0.01). These estimates indicate that an individual with plasma PUFA index of 200 ug/ml is expected to complete the Trails B task a total of 4 seconds earlier by comparison with an individual with a PUFA index of 100 ug/ml for each year increase in age. Given that each year increase in age was associated with a mean Trails B completion time of 4 seconds longer over follow up, these estimates predict a one year delay in the decline of executive function attributed to age per 100 ug/ml increase in the PUFA index.

**Table 2**. PUFA index (EPA+DHA) is associated with less executive decline in non-demented elders over 4-years follow up					
		**Baseline function** (Intercept)		**Cognitive decline** (Slope)	
		β (SE)	***P***	***β (SE)***	***P***
1	Trails B	-0.73 (0.27)	0.01	-0.04 (0.02)	0.01
2	Digit Symbol	0.05 (0.00)	0.24	0.01 (0.00)	0.07
3	Paragraph Recall	0.02 (0.02)	0.26	0.00 (0.00)	0.40
4	MMSE	0.01 (0.01)	0.42	0.00 (0.00)	0.24

<u>Study 2</u>: The effect of PUFA levels on executive function is mediated by white matter hyperintensities. Since the PUFA index is correlated with WMH (figure 3), we performed a mediation analysis to clarify the relationships between PUFAs, WMH, and decline on Trails B. The association between PUFA index and annualized decline in Trails B was re-established in the subset with WMH available (Table 3, model 1). Total WMH also associated with accelerated executive decline (Table 3, model 2). After inserting PUFA index and WMH as simultaneous predictors of Trails B change in the final step, we observed the PUFA index no longer associated with Trails B while WMH remained a significant predictor of accelerated executive decline (Table 3, model 3).

**Table 3**. PUFA and cerebrovascular pathology mediated executive decline			
	Predictor	Trails B decline (Slope)	
		β (SE)	P
1	PUFA index	- 0.05 (0.02)	0.03
2	WMH	4.78 (1.39)	0.00
3	PUFA index<br>WMH	-0.01 (0.04)<br>4.86 (2.16)	0.89<br>0.02
PUFA index adjusted for baseline PUFAs and WMH for intracranial volume			

<u>Study 1 and 2 results suggest that pufas decelerate executive decline in non-demented elders by mechanisms involving less WMH accumulation</u>

<u>Study 3</u>: Plasma ICAM-1 predicts rate of decline in cognitive function (Table 4) and rate of increase in WMH (Table 5):

<u>Study 4</u>: Plasma pufas are inversely associated with plasma soluble ICAM-1 in OBAS cohort. Figure 4 shows the inverse relationship between plasma pufas and plasma ICAM-1. The cross-sectional linear regression analysis estimate suggests that each 100 ug/mL increase in plasma PUFA index (EPA+DHA) is associated with a modest 0.3 ng/mL decrease in plasma ICAM-1 (*p* = 0.02, figure 4A). Plasma EPA appears the most significant to plasma ICAM-1 (figure 4B).

Figure 3. Cross sectional association between PUFA index and total WMH volume.

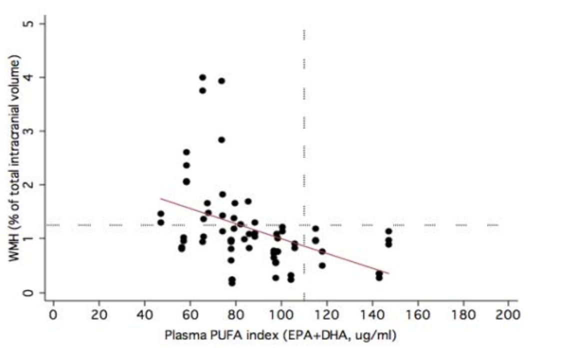

Vertical reference line: PUFA index of 110 ug/ml (or 5% of total), horizontal line: 1.25 cm3 of total WMH. OBAS subjects with MRI were older (mean age=92.4 ±3.5, range 85–99) compared to those without MRI

**Table 4**. Plasma ICAM-1 associated with accelerated cognitive decline in OBAS*					
		Baseline function (Intercept)		Cognitive decline (Slope)	
		β (SE)	*P*	*β (SE)*	*P*
1	Trails B	0.042 (0.087)	0.63	0.015 (0.002)	<0.001
2	Digit Symbol	-0.014 (0.015)	0.33	-0.003 (0.000)	<0.001
3	Paragraph recall	0.004 (0.007)	0.57	-0.001 (0.000)	<0.001
4	MMSE	0.003 (0.003)	0.38	-0.001 (0.000)	<0.001
*Mixed effects models adjusted for baseline plasma ICAM-1					

**Table 5**. Plasma ICAM-1 associated with accelerated WMH progression in ADNI**					
	Predictor	Baseline WMH (Intercept)		WMH progression (Slope)	
		β (SE)	*P*	*β (SE)*	*P*
1	ICAM-1	-0.568 (0.491)	0.25	0.013 (0.004)	0.001
Mixed effects models adjusted for baseline plasma ICAM and total intracranial volume,<br>**Alzheimer's Disease Neuroimaging Initiative, AD subjects excluded, n=404					

Figure 4. PUFAs and ICAM-1

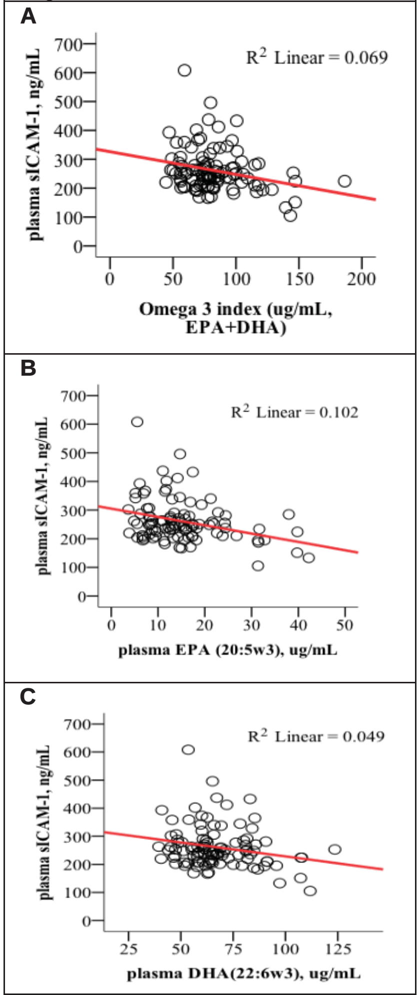

Study 3 and 4 results suggest that endothelial dysfunction is a risk factor for cognitive decline and white matter disease, and raise the possibility that pufas modulate cerebrovascular function by a mechanism involving ICAM-1

Refined hypothetical model for how pufas promote brain health

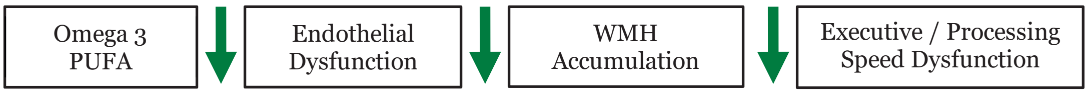

***Study 4*: Pilot randomized trial in Alzheimer's disease confirms the bioavailability and safety of the proposed omega 3 pufa preparation over 1 year**.

This NIA funded (R21AG023805) trial conducted by Drs. Lynne Shinto and Joseph Quinn at OHSU tested the bioavailability, safety, and efficacy of pufas and pufas (fish oil extract) + lipoic acid in slowing AD progression. Enrolled subjects were age 55 and older with NINCDS/ADRDA defined AD, a MMSE of 15-26, and CDR ≤1.0 (Table 5). PUFA supplementation 4 weeks prior to study enrollment or fish intake of 6 ounces or more/week within a month of study evaluation disqualified patient entry. Thirty-nine subjects were enrolled and randomized to a placebo (n=13), fish oil only (n=13) or fish oil + lipoic acid (n=13, data not shown). Three fish oil capsules, each 23.5 mm long containing a gram of oil concentrate with 325 mg of EPA and 225 mg of DHA each providing a total of 1.65 g of pufas (975 mg EPA and 675 mg DHA) (ProOmega, Nordic Naturals, Watsonville, CA). Three soybean oil capsules of a gram each with same appearance and taste as the active capsules were administered daily as a control. ***Results:*** Eighty-seven percent (34/39) of the participants completed the study at 12 months. The bioavailability and safety data for the pufa preparation (fish oil) and placebo are provided in figure 6 and Table 6**.** Both EPA and DHA levels were higher in the fish oil arm compared to placebo at 6 and 12 months (p<0.001) whereas the soybean oil placebo had no such increase in pufa levels at 6 or 12 months compared to baseline. ***The fish oil raised the percentage of EPA in RBC's by 2.5% (0.5% to 3.0%) and RBC DHA percentage by 3.5% (4.4% to 7.9%) at 6 and 12 months (figure 6)***. Adverse events were monitored monthly. ***The fish oil was remarkably well tolerated in this older population (Table 6).*** Most AE's were mild and there were no significant differences between groups in number of AE reports or types of AE reported. There were two serious adverse events (death) not related to the study medication, one in the placebo group and one in the pufa group. ***Compliance*** was monitored by in-clinic pill count every 3 months. Mean compliance range was between 79.4% to 97.8% for all groups at 6 and 12 months and there were no significant differences between

Table 5. Baseline Characteristics
(All *P* values for difference between groups > 0.05)

	**Soybean oil** (n=13) Mean (SE)	**Fish Oil** (n=13) Mean (SE)
Age	75.2 (10.8)	75.9 (8.1)
Female	54%	39%
White	100%	100%
College	54%	39%
BMI	23.8 (3.1)	26.2 (4.5)
MMSE	22.2 (3.1)	20.7 (2.7)
RBC DHA (%)	4.4 (1.0)	5.1 (1.3)
RBC EPA (%)	0.55 (0.14)	0.60 (0.23)
*AD med use	77%	92%

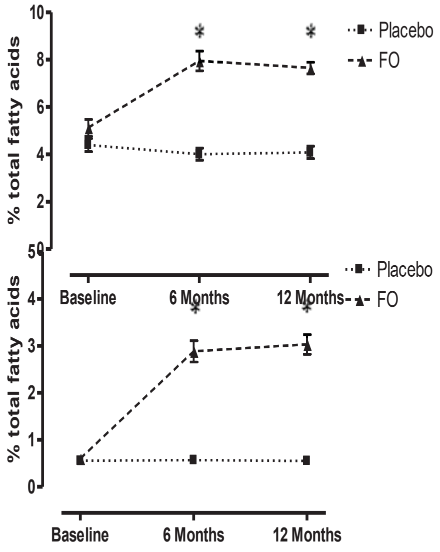

Figure 6: Bioavailability of ProOmega 3 Fish Oil (FO) versus placebo: RBC membranes are enriched with EPA and DHA in FO group at 6 months and 12 months (*p < 0.05)

**RBC EPA**	**RBC DHA**

Table 6. *Adverse Events*

	**Placebo** (n=13)	**Fish oil** (n=13)	**Fish oil + lipoic** (n=13)	***P****
Cold or Flu	3	2	2	0.84
Loose stools	3	0	3	0.17
Dizziness	1	2	2	0.80
Falls	2	1	0	0.34

*P-value reflects a comparison of all groups for each type of AE reported

groups in compliance for fish oil/fish oil placebo or lipoic acid fish oil/lipoic acid fish oil placebo at any time points.

***Summary of the Preliminary Studies Leading to the Primary Hypothesis*.** Plasma pufas are inversely associated with WMH volume in non-demented elders; they are also associated with age-related decline in executive function and processing speed and this appears to be mediated by WMH. The pufas explain 28.5% of the total variation in total WMH volume. Blood-based biomarkers of endothelial function (e.g. ICAM-1) are associated with accelerated rate of cognitive decline and WMH progression. PUFAs are inversely associated with plasma ICAM-1, suggesting they may attenuate endothelial dysfunction that we show is significant to brain health. We have experience with the proposed pufa supplement, including its bioavailability and remarkable safety profile in elders. In summary, this body of evidence supports the hypothesis that cerebral small vessel disease and endothelial function are the "locale" for pufa action. These and existing studies justify our emphasis on total WMH, endothelial function and measures of executive function and processing speed skills as the earliest signals of clinical relevance for pufa effects.

Overall Objective. The overall objective is to determine if the intervention will prevent late life cognitive decline and dementia, utilizing a reliable and valid biomarker of cerebrovascular disease progression and dementia risk. More concretely, the primary study objective is to determine the effects of pufa supplementation on MRI-derived brain WMH progression in non-demented elders followed for 3 years (Aim 1). Our primary hypothesis is that subjects randomized to pufas will appreciate less total WMH accumulation over the study duration compared to placebo. Another objective is to determine the effects of pufa supplementation on biomarkers of endothelial function over 3 years (Aim 2). The primary hypothesis for Aim 2 is that subjects randomized to pufas will appreciate lower plasma ICAM-1 compared to placebo. We will also explore other biomarkers related to inflammation (VCAM-1, E-selectin, IL-6, TNF-alpha, hsCRP), amyloid (a-beta 40 and 42 amino acid length) and lipid fractions. The third objective is to collect preliminary trial data on the pufa effects on psychometric indices that appear most sensitive to the accumulation of WMH (i.e. executive function).

Experimental Design. The design is based upon the observation that WMH volume and advanced age are strong predictors of WMH progression and cognitive decline in non-demented elders. The single-center, randomized, double-blind, placebo controlled clinical trial will test the efficacy of pufas in slowing WMH progression, improving blood-based biomarkers of endothelial function, and slowing decline in executive function and processing speed. The study population consists of non-demented elders age 75 and older who also meet entry criteria for lower plasma pufa status and higher WMH burden at baseline. The intervention is a 50:50 randomization to an active standardized pufa formulation (fish oil extract) vs. a standardized placebo soybean oil over a 3 year period. The pufa formulation and dosage are based on our pilot study and experience with this formulation showing an excellent safety profile and predictable blood concentration. Total WMH volume will be evaluated at entry and annually to analyze the primary outcome. Serially collected plasma samples will be analyzed at baseline, 3 months, 6 months, and then every 6 months to confirm compliance and examine biological activity of the pufas. Neuropsych test emphasis is placed upon domains known to associate with WMH accumulation and pufas and will be collected at baseline and annually. Clinical Dementia Rating-Sum of Boxes and Activities of Daily Living will also be collected in order to determine if there are any more clinically significant effects of the intervention, although we recognize that cognitive and functional change in this relatively healthy population is very modest in this time frame.

Study Population

Inclusion Criteria

1. Non-demented or mild cognitive impairment, defined as Clinical Dementia Rating =0 or 0.5 and MMSE >=24.
2. Age 75 and older, male and female
3. Total WMH volume ≥ 5 cc
4. Plasma PUFA index (EPA + DHA) < 110 ug/ml or < 5.5 weight percent
5. Sufficient English language skills to complete all tests
6. Geriatric Depression Scale – 15 < 6 documenting absence of a significant depressive syndrome
7. Sufficient vision and hearing to complete all tests
8. Informant available with frequent (at least 1 hour/day or 1 day/week) contact with subject to verify functional status and CDR rating
9. General health status that will not interfere with the ability to complete the prospective study (these conditions are listed below in the study exclusion list)

Exclusion Criteria

1. Any dementing illness (AD, vascular dementia, normal pressure hydrocephalus, or Parkinson's disease); dementia defined by CDR ≥ 1, MMSE < 24
2. Significant disease of the CNS such as brain tumor, seizure disorder, subdural hematoma, cranial arteritis
3. Alcohol or substance abuse according to DSM-IV criteria within the last 2 years
4. Major depression, schizophrenia, or other major psychiatric disorder defined by DSM-IV criteria
5. Abnormal labs indicating vitamin B12 deficiency, thyroid disease, or UTI (documented bacterial colonization is acceptable)
6. Unstable or significantly symptomatic CVD (e.g. CAD with frequent angina, CHF with dyspnea at rest)
7. Hypertension: defined as uncontrolled BP > 150/90
8. Clinical symptomatic orthostatic hypotension
9. Diabetes mellitus that requires insulin injections
10. History of cortical stroke
11. Cancer within the last 5 years, with the exception of localized prostate cancer (Gleason Grade < 3) and non-metastatic skin cancers (melanoma).
12. Illness that requires >1 visit /month to a clinician

13. Contraindications to MRI (i.e., heart pacemaker, metal plates or objects in head, , claustrophobia)
14. Medications:

    a. CNS active meds that have not been on stable doses for at least 2 months (cimetidine, beta-blockers, and SSRIs)
    b. Neuroleptics, antiparkinsonian agents, systemic corticosteroids, and narcotic analgesics; in the case where these were used for a self-limited time they must have been discounted for a period of five half-lives prior to baseline visit
    c. Over the counter supplements are not by themselves exclusionary, however, subjects are asked not to change the dosing regimen over the course of the trial unless medically indicated; the presence and dose of these agents are recorded
    d. A baseline screen plasma PUFA > 5.5 weight percent of total fatty acids for EPA+DHA will confirm supplementation of O3PUFA history. If patient indicates regular supplementation with fish oil on phone screen, can wash out for 4 months prior to study visit one.
    e. Cholinesterase inhibitors (i.e., Aricept)
    f. Investigational drugs within five half-lives prior to baseline
    g. Anticoagulation therapy: Vitamin K antagonist: warfarin (Coumadin, jantoven), Factor Xa inhibitors: rivaroxaban (xarelto), fondaparinux (arixtra), dibigatran (pradaxa), apixaban (eliquis); Low molecular weight heparins: dalteparin (fragmin), enoxaparin (lovenox)(Incident use of anticoagulant therapy will exclude further study drug allocation. However, subjects will be asked to complete all follow-up visits.)

Study Supplement and Dose. Because neuronal functioning is dependent on DHA [66] and EPA may have vascular effects [67], we have elected to use a combination supplement. Since these pufas also exist together in nature, this form of supplementation will be less likely to create an unfavorable imbalance in fatty acid metabolism compared to a single constituent form, especially during long-term use in an older population. The form and daily dose of EPA and DHA was chosen because it is a form used in a previous randomized, double-blind, placebo-controlled study in elders with mild to moderate AD (Table 5). This dose was able to significantly increase both EPA and DHA levels in RBC membranes at 6 and 12 months in study participants randomized to receive fish oil (n=13 at both time points), and was associated with an excellent safety profile (Figure and Table 6). This dose also penetrates the threshold plasma levels associated with neuroprotection in our preliminary and published studies. The study supplement will contain one of the following and all study supplements will be taken with meals: 1) Each ProOmega 3 (Nordic Naturals) soft gel contains 1 gram of fish oil concentrate guaranteed to have a concentration of long-chain omega 3 fatty acid ethyl esters at 70%, primarily EPA (325 mg/g) and DHA (225 mg/g). Three capsules is the daily dose, which will provide a total of 1650 mg of omega 3 pufas (975 mg EPA + 675 mg DHA) per day; 2) Placebo oil contains soybean oil.

Outcome Measures. Measures of executive function and processing speed appear sensitive to WMH progression[18]. Our preliminary data found plasma omega 3 pufas associated with WMH volume and age-related decline in domains known to be sensitive to WMH accumulation (Trail B and Digit Symbol). We intend to use the same psychometric measures to determine omega 3 pufa supplementation effects. An axial view of a 3T FLAIR MRI will be utilized at screening to determine total WMH volume and permit entry of subjects with 5 cc or >. MRI derived total WMH volume is a primary outcome measure. The Clinical Dementia Rating scale will confirm non-demented state as CDR < 1.

Recruitment and Enrollment: A de-identified scan of the OHSU clinical database identified 6,571 men and women age 80 and older that have visited our medical center within the past year, without a dementing illness, contraindication for MRI (pacemaker), without ventricular-tachycardia or v-fib, IDDM, cancer and reside within reasonable proximity to OHSU (Oregon and Washington counties close to Portland) and Oregon (counties include Multnomah, Clark, Clackamas, Washington, Columbia, Polk, Marion, Deschutes) and are eligible for recruitment. Assuming 20% of these elders have no evidence of WMH burden and plasma EPA+DHA above 110 ug/mL, 5,256 elders remain potential study participants. To meet our target of 150 subjects enrolled at baseline, we would need to recruit <3% of this population. In addition, the NIA-Layton Aging & Alzheimer's Disease Center maintains direct contact for its ongoing longitudinal aging studies in over 50 aging centers, retirement communities and community organizations in the Portland metro area. Research Data Warehouse at OHSU will identify people with a profile making participation possible. Partnership with the Vancouver Clinic (TVC) has been established for study recruitment. TVC has conducted an EPIC Query and identified ~3,200 patients who match our inclusion criteria. TVC has agreed to release this data to OHSU after sending an opt-out letter to potential participants. Those who do not respond to the opt-out letter in two weeks' time will be contacted by the OHSU research staff.

Description of study visits **(see Study Event Schedule below)**
The MRI, cognitive and other behavioral, functional, and motor measures are collected annually. We will collect blood based biomarkers at month 3 and 6 in the initial year of participation to examine short-term changes during therapy. Blood-based biomarkers are collected every 6 months after the initial year is complete. All study visits where blood draws are performed for screening, safety, and blood-based biomarkers will be fasted. For participants who express extreme anxiety about undergoing an MRI scan, one of the co-investigators licensed to prescribe medication may prescribe a mild anti-anxiety medication to be used for these isolated instances.

Pre-screening (in clinic or by phone). Prior to an in-person screening, potential participants are identified by the investigative group through routine clinical contact or through a pre-screening process. Prospective subjects will be non-demented and known to meet as many of the inclusion/exclusion criteria as possible. These subjects will attend a clinic screening with a study partner and bring medications that they are currently taking. If study partner is unable to attend clinic screening, a phone interview will be conducted with the study partner, and consent for this will be obtained over the phone. Study subjects will not be considered study participants until they attend the in-clinic screening and clearly relay their understanding of the study protocol, corroborated verbally and through signed informed consent. **If a participant lives over 100 miles from OHSU, or has significant impairment that makes traveling to OHSU difficult, then we will provide the option for the participant to come to OHSU for a combined visit that consists of both aspects of visits 1 and 2.**

Screen I (visit 1). Informed consent will be obtained from both the study subject and the study partner when they arrive at the clinic. Demographics and general medical history will be obtained including psychiatric history, family history and history of allergies. A general physical and neurological exam will be performed including vital measures. To accommodate participants' schedules, the general physical and neurological exam may be performed at Screen II (visit 2) instead. A review of pre-study medications for up to 30 days prior to screening will occur. A non-dementia status will be confirmed using the same diagnostic protocol used in the Oregon Alzheimer's Disease center. The MMSE, CDR, GDS, screening labs, safety labs, and the addition of a plasma PUFA index (EPA + DHA in ug/mL or % <5.5% total fatty acids) will be performed. This information will be used to establish the eligibility as outlined under protocol Inclusion/Exclusion criteria. Screen I/Visit 1 will take approximately 2 hours. **If visits 1 and 2 are combined, the participant will be asked**

to come in fasted for their appointment, and study staff will wait to randomize the participant pending meeting all inclusion criteria.

Screen I Fail Procedure: Participants who are ineligible for study will be notified and provided an explanation for their ineligibility. The total number of screen failures and justification will be documented. Plasma PUFA index failures are counted and documented.

Screen II (visit 2). MRI will identify subjects with high WMH. MRI screen is performed within 56 days of the Screen I (visit 1). This brain scan determines WMH eligibility as outlined in the protocol Inclusion / Exclusion criteria. This visit will take approximately 3 hours. Participant will also complete a Food Frequency Questionnaire administered by a nutritionist in clinic to answer questions and assure complete data, and central blood pressure will be measured by a research coordinator. The general physical and neurological exam will also be completed if not done at Screen I (visit I).

Screen II Fail Procedure: Participants who are ineligible for study will be notified and provided an explanation for their ineligibility. The total number of screen failures and justification will be documented. WMH volume failures are counted and documented.

Baseline and Randomization (visit 3). Subjects meeting the inclusion criteria will participate in a baseline visit within 56 days of Screen I (visit 1) and will be assigned a sequential number determining group assignment. The statistician (Dr. Hiroko Dodge) will generate the randomization sequence. Age, sex, levels of education, WMH volume and CDR will be balanced between the two groups using the modified randomized minimization algorithm[68]. At the baseline visit, behavioral/cognitive, functional and motor assessments will be obtained with the Wechsler Adult Intelligence Scale (WAIS-IV) Digit-Symbol Coding Section, Trail Making Test – Part A then Part B, Craft Story 21 Recall–Immediate, Benson Complex Figure Copy- Immediate, Montreal Cognitive Assessment (MoCA) , Number Span Test (Forward & Backward), Multilingual Naming Test (MiNT), Craft Story 21 Recall–Delayed, Benson Complex Figure Copy- Delayed, Category Fluency (Animals & Vegetables), Letter Fluency (C & L), ADLs, and Gait Speed. Vital signs, ECG, and blood-based biomarkers (including DNA and RNA) are collected. Pre-existing conditions and concomitant medications are recorded; a 3-month supply of the study supplement is dispensed after randomization sets allocation (after visit 2). A 3-month supply of study medication will be dispensed at this visit with verbal and written instructions. This visit will take approximately 2 hours.

Month 3, 6, 18, 30 (visit 4, 5, 9, 13). This clinic visit will record adverse events and concomitant medications. The blood-based biomarkers and vitals will be collected. Study medication will be assessed for compliance and dispensed. At months 6, 18, and 30 (all but month 3), a physical and neurological examination will be conducted and safety labs will be collected every 6 months until month 24 where safety labs are only collected at month 36 thereafter unless abnormalities are identified at month 24.. RNA will be collected.

Months 9, 15, 21, 27, 33 (visit 6, 8, 10, 12, 14). These phone visits will record adverse events and concomitant medications. Study supplement will be assessed for compliance and dispensed.

Month 12, 24, 36 (visit 7, 11, 15). These clinic visits will collect MRI, Behavioral/Cognitive, and Functional measures. Vitals, a physical and neurological exam, safety labs and blood-based biomarker will be completed. Adverse events and concomitant medications will be recorded; study medication will be assessed for compliance and dispensed. At the month 36 visit, the participant will complete the Food Frequency Questionnaire administered by a nutritionist. At month 12 an ECG and RNA will also be collected. These visits will take approximately 5-6 hours. If the participant has a significant impairment that makes coming to OHSU for an extended time too difficult, we will provide them the option to split the visit between two days. The visit will be divided as such that the MRI will take place on a day separate from behavioral/cognitive testing and functional measures. The two visits will occur within 28 days of each other.

Table 7. Study Event Schedule

Visit number	1	2	3	4	5	6	7	8	9	10	11	12	13	14	15
**Visit name/Month**	**Screen 1**	**Screen II**	**Base**	**3**	**6**	**9**	**12**	**15**	**18**	**21**	**24**	**27**	**30**	**33**	**36**
Consent	X														
Demographics/Medical history	X														
Medications	X		X	X	X	X	X	X	X	X	X	X	X	X	X
Food Frequency Questionnaire		X													X
Vitals	X		X	X	X		X		X		X		X		X
MMSE	X						X				X				X
Clinical Dementia Rating-SOB	X						X				X				X
Geriatric Depression Scale	X						X				X				X
Physical and neurological	X				X		X		X		X		X		X
ECG			X				X								
Screening labs[2]	X														
Safety labs[3]	X				X		X		X		X				X
MRI		X					X				X				X
Neuropsych/Functional Measures			X				X				X				X
Blood-based biomarkers[4]			X	X	X		X		X		X		X		X
DNA			X												
RNA			X	X			X								
Dispense meds/Randomization			X	X	X	X	X	X	X	X	X	X	X	X	
Blinding evaluation															X
Compliance/Pill count				X	X	X	X	X	X	X	X	X	X	X	X
Adverse events				X	X	X	X	X	X	X	X	X	X	X	X

[1]NCI–Food frequency questionnaire; [2]CBC, CMP, TSH, B12, EPA+DHA for entry; [3]CBC, CMP, PT/INR; [4]Fatty acids, endothelial function markers (sICAM-1, sVCAM-1, E-selectin), inflammatory (TNF-alpha, IL-6, hsCRP), amyloid metabolites, lipid fractions

Blinding and Randomization. This is a blind placebo controlled study. Based on passing all screening criteria, subjects will be assigned to treatment group using a modified randomized minimization algorithm [68]. The placebo group will be matched to the experimental group in the following factors: age group (5 year categories), sex, education, global CDR scores and WMH volume. The participants, study investigators, research associates, and study coordinators will have no knowledge of study assignment. Data analysis will be performed blinded to treatment status. The project biostatistician (Dr. Dodge) will create the randomization scheme and will ensure blinding of all study medications and data records. Study medication will be handled, labeled, and distributed by pharmacists in Research Pharmacy Services (RPS) who are independent of the study otherwise. We will also evaluate the effectiveness of our blinding by giving study evaluators, subjects, study partners, and investigators a short questionnaire asking about knowledge of group assignment. The randomization code will be broken only after data analysis or if there is an unusual circumstance, such as numerous serious adverse events before the end of the study.

Adverse events. Adverse events will be monitored in "real time" with weekly review of AE's with study staff using clinical judgment to determine reporting to IRB and FDA. An external data safety monitoring board (DSMB) will convene biannually to study unblinded data on AE coded by organ system. Although assessment of AEs will also include solicitation of non-specific change (e.g., "do you feel different since beginning the treatment?"), a 12-item AE checklist covering all major organ systems will be included to probe for AEs. The nature of each AE, its severity (mild, moderate, or severe), its likely relationship to study treatment (definite, probable, possible, not related, or unknown), its duration, and any necessary treatment modifications or adjustments will be recorded. In addition to the monitoring and recording of AEs at each 3 month subject contact, labs to assess basic metabolic function (including liver function tests), CBC and PT/INR are performed at screening and at months 6, 12, 18, 24 and 36. Incident use of oral anticoagulant therapy is identified through subject interview that occurs every 3 months. Incident anticoagulation therapy cases are asked to stop the supplement and complete complete the remaining follow up visits. Participants will be reminded and encouraged during clinic visits and phone checks to contact the study coordinator or investigator if a moderate or serious AE occurs.

Compliance: Compliance will be measured in-clinic by pill count. In addition to the in-clinic checks on compliance, each subject will receive a phone call to check on compliance every 3 months. If a subject's compliance drops < 80% at any time, they will be contacted by the trial coordinator by phone to find out why

and to aid the subject in finding ways to increase compliance. Although plasma fatty acid measurement is an independent measure of compliance, knowing the results of fatty acids levels during the study would un-blind the study. Plasma PUFA index will be used during data analysis to corroborate pill count.

***Statistical Analysis*:** **Aim 1: Determine pufa effects on WMH accumulation over 3 years in non-demented elders, age ≥ 75 with low plasma pufa status and WMH burden at entry.** This aim will test the hypothesis that pufas slow WMH accumulation compared to the placebo. **Aim 2: Determine pufa effects on biomarkers of endothelial function.** This aim will test the hypothesis that pufas improve plasma measures of endothelial function . **Aim 3: Determine pufa effects on cognitive change.** This aim tests the hypothesis that pufas slow the executive function and processing speed decline, an effect mediated by WMH.

Analytic approach. First we will conduct univariate analyses using changes in WMH progression (**Aim 1**), ICAM-1 (**Aim 2**), and cognitive test scores (**Aim 3**) between baseline and three follow-up assessments (baseline, 12, 24 and 36 month) for WMH, ICAM, and cognitive outcomes with a predictor variable being pufas active vs. placebo. Student's t-test will be used for this descriptive analysis based on ITT. Next we apply linear mixed effects multiple regression models with longitudinal WMH progression (**Aim 1**), sICAM-1 (**Aim 2**), and cognitive test scores (**Aim 3**) as outcomes, a main predictor variable as a dummy variable for pufas (active vs. placebo as a reference) and its interaction with a time variable. Control variables include age at baseline, sex, past history and incidence of vascular diseases including hypertension, ApoE4 allele and depression. Intercept and time variables in years (years from baseline) are treated as random effects in all models. We will use an unstructured error covariance structure and estimate parameters using restricted maximum likelihood procedures. Missing data points in the analytical sample are considered missing at random[69] and the above approach is adequate under this assumption. Interaction term between the pufa and placebo groups and time is of our substantive interest. That is, we are interested in examining the magnitude and significance of the difference in time slopes between the active and placebo groups. In all models quadratic and higher order time variables will be included if it improves the model fitness. The overall fit of the models will be examined using a combination of formal fit criteria and visual inspection of residual plots. Results will be considered significant at $p < 0.05$, but we will also provide Bonferroni-adjusted p-values to correct for multiple statistical tests.

Sample size calculations:

<u>Attenuated rate of total WMH progression (**Table 8**)</u>: The sample size calculation is based upon the information obtained from Oregon Brain Aging Study. Assuming a 40% reduction in WMH progression over 3 years among the active group, we would have 80% power to detect this difference with a sample size of 50 subjects per arm at alpha = 0.05 (two-tailed). Given that we may have 50% reduction in WMH progression over this period, we would achieve 80% power to detect this difference with a sample size of 32 subjects per arm at alpha=0.05 (two tailed). Thus, with a conservative estimate of about 30% attrition over 3 years, we will achieve at least 80% power to detect reasonable effect size (40%) with a sample size of 150 subjects at baseline.

Table 8. Sample size calculations for mean change in WMH progression over 3 years*

Power 0.80	Effect size (%)	Difference in change between pufa and placebo	Placebo: Mean (SD) changes	PUFA: Mean (SD) changes	Sample size needed per arm
	40	2.8	7.0 (5.0)	4.2 (5.0)	50
	50	3.5	7.0 (5.0)	3.5 (5.0)	32

*Derived from Oregon Brain Aging Study cohort data in participants age 80 and older with total WMH volume ≥6 cm^3

<u>Slowed rate of plasma ICAM increase</u>: Our preliminary data demonstrates that the mean plasma ICAM in non-demented participants of the OBAS under the age of 80 and those age 80 and older were 219.19 ng/mL (SD 39.12) and 269.34 ng/mL (SD 78.89), respectively. We expect that in our trial population (all age 75 and older) a reduction of plasma sICAM-1 in the active group will achieve plasma pufa levels of that observed in the younger participants of OBAS (<80 years old). Under this scenario, we would have over 97% power to detect a reduction in plasma ICAM of this magnitude with the proposed sample n=50 for each arm (i.e., about 52 each at the end of the 3rd year assessment and after 30% drop out with 150 enrolled at baseline).

<u>Slowed rate of Trails B and Digit Symbol change</u>: This exploratory aim assumes an annual rate of change in Trails B of 4 seconds and Digit Symbol score of 0.75 in subjects 80 and older (derived from OBAS data) and a total 3-year change of 12 (±13) and 2.25 (±2), respectively. With 50 subjects in each arm at the end of 3 years follow-up, we would have 80% power to detect the standardized effect size of 0.6 (for example, trails b change

scores: 12 (placebo) vs. 4.7 (active), digit symbol: 2.3 (placebo) vs. 1.1 (active). The distribution of changes in neuropsychological test scores obtained in this pilot trial will provide the basis for the future larger study.

Description of Measures:

MRI data acquisition: will be obtained using the same 3T MRI instrument using phased array RF coils housed in the OHSU/Advanced Imaging Research Center (AIRC). Subjects will undergo anatomical imaging sequences similar to those used in the ADNI[70]. We will use the following protocol: 1) *T1-weighted magnetization prepared rapid gradient echo (MPRAGE)*: TE = 3.4 ms; TR = 2300 ms, TI = 1200 ms; 1 mm isotropy; full brain coverage; FOV 256 mm; 2) *T2/Proton density (PD) duel fast spin echo*: TE = 11 ms, TR = 3,000 ms, slice thickness = 3 mm in-plane resolution 0.9 mm, 3) *Fluid attenuated inversion recovery (FLAIR)*: sagittal, TE = 388 ms; TR = 6,000 ms, TI = 2,100 ms; 1 mm isotropy; full brain coverage; FOV = 250 mm, and 4) *Diffusion tensor imaging (DTI)*: TR = 9500 ms; TE = 95 ms; slice thickness 2.0 mm; 30 directions. Total MRI acquisition time will be less than one hour. Imaging data will be stored in a secure server located in the AIRC at OHSU. In order to maintain scanner stability over time, ADNI quality assurance measures (i.e., calibration phantom and retrospective image correction) will be implemented, 4) *Arterial spin labeling (ASL)*, 5) Hemorrhage sequence.

MRI Data analysis:

Subject inclusion criteria: Total WMH volume: For the purpose of enriching a study population with enhanced WMH burden, a preliminary determination of WMH severity will be assessed using FLAIR images[71]. At the screen II visit, axial FLAIR MRI images will be quantified to determine high total WMH (≥ 5 cc).

MRI Primary Outcome: Volumetric total WMH segmentation: Freesurfer tissue segmentation is performed on the T1 images. FLAIR images are affined registered to T1 sequences using FLIRT[72] with 6 df and masked by WM tissue masks from Freesurfer. The mean and SD of intensities within the WM mask are calculated with AFNI tool (3dMaskAve). Clusters of voxels over 2.5 standard deviations above the mean in intensity are used as seed voxels for a cluster-growing algorithm[73]. A customized matlab script calculates the mean signal intensity within each cluster and iteratively incorporates all nearest neighbor voxels that are within 5% signal intensity and within the WM mask. Approximate voxels with similar intensity are added to each cluster, the mean is recalculated, and the process repeats until surrounding voxels of similar intensity are absent. Clusters of contiguous WMH voxels, which extend to the border of the lateral ventricle, are automatically labeled periventricular while all other clusters are labeled deep. This WMH segmentation method is validated in house against WMH volumes obtained by manual tracings (R^2 = 0.92, plot not shown). The fully automated method produces excellent intra-rater (ICC = 0.99) and inter-rater (ICC = 0.99) reliability.

MRI Secondary Outcomes: 1) Voxel-Based Morphometry (VBM) analysis uses Statistical Parametric Mapping (SPM) software, normalized and segmented in Montreal Neurological Institute (MNI) stereotactic space is used to examine regional differences in grey matter volumes between the active and placebo groups 2) Volumetric Brain MRI will use Freesurfer to determine the group differences in medial temporal lobe volume changes, which is another region that may be sensitive to pufas[74-76]. 3) DTI and TBSS will examine regional, between group differences in white matter tract integrity previously linked to pufa supplementation[77]. Voxel wise statistical analysis of FA data is performed using TBSS[78] and FSL[72]. FA images are created by fitting a tensor model to the raw diffusion data using FSL's diffusion toolbox, and then brain-extracted[79]. FA data is then aligned into a common space using the nonlinear registration tool FNIRT. Mean FA image is thinned to create a FA skeleton representing the center of all tracts common to the group. Each subject's aligned FA data is then projected onto this skeleton and the resulting data is fed into voxel wise cross-subject statistics. Mixed effects models determine time point, group and interaction effects. 4) ASL will examine cerebral blood flow.

Biomarkers and Diet Survey. Plasma fatty acids are measured using GC/MS, soluble adhesion molecules and inflammatory products (sICAM-1, sVCAM-1, E-Selectin, TNFα, IL-6, hsCRP), and beta amyloid metabolites (abeta 40 and 42) with ELISA, enzymatic methods for lipids, PCR for APOE4 in the OHSU/Oregon Clinical & Translational Research Institute's Core Laboratory[2]. For plasma PUFA (EPA + DHA) < 5.5% total fatty acids at screening visit 1, the Homan Omega 3 blood spot test will be used as this test allows for a 10 day turn-around of results, ensuring that we will meet all baseline inclusion/exclusion criteria in a timely fashion[92,93].These measures (except APOE4) are collected at baseline, 3, 6, 12, 18, 24, 30 and 36 months.

The Food Frequency Questionnaire is collected at baseline and at month 36 to assess stability of diet during the course of the study[80] and assess any dietary drift within treatment groups.

Neuropsychological assessment (Admin total time ~ 45 minutes)

Trail Making Test: *Trails A*: number sequence administered to familiarize participants with the task. *Trails B*: Investigates speed of attention, sequencing, mental flexibility, visual search and set shifting. Participants are asked to join 25 randomly displayed numbers and letters in an alternating and sequential fashion.[81] [Admin 10 minutes]*Digit Symbol Test*: The WAIS-IV Digit Symbol Test is a visuographic-coding task in which participants code numbers into symbols using a key. Speed is scored[82]. [Admin 3 mins]

Category Fluency (Animals & Vegetables): Subject is asked to name, as fast as they can, all of the things that belong in a given category. Subjects will be given 60 seconds to name items in the category of Animals, and an additional 60 seconds to name items in the category of Vegetables. [Admin. 5 mins]

Letter Fluency (C & L): Subject is asked to name, as fast as they can, all of the words they can think of that begin with certain letters. Subjects will be given 60 seconds to name words that begin with the letter "C," and an additional 60 seconds to name items that begin with the letter "L." [Admin. 5 mins]

Multilingual Naming Test (MiNT): Participants are shown a picture of an object, one at a time, then asked to name the object. Participants are scored for correct responses, as well as prescribed prompts. This test is designed to assess naming skills. [Admin. 5 mins]

Craft Story 21 Recall: This test assesses memory. Participants are asked to repeat back a short story. Participants are scored on verbatim responses and separately on paraphrased responses. Participants are asked to recall this story again after a 20 minute delay. [Admin. 10 mins]

Number Span Forward & Backward: This test assesses attention and short-term memory. Participants hear a list of numbers and must repeat them in the same order. Participants will also hear a separate list of numbers and be asked to recount them in reverse order. [Admin. 10 min.]

Benson Complex Figure Copy: This task assesses memory and visuospatial processing. Participants view a complex design and are asked to replicate it, and then do so again after a 15 minute delay. [Admin. 10 mins]

Order of administration: WAIS-IV Coding, MoCA, Craft Story 21 Immediate Recall, Benson Complex Figure Copy Immediate Recall, Number Span Forward, Number Span Backward, Category Fluency (Animals & Vegetables), Trail Making Test A, Trail Making Test B, Craft Story 21 Delayed Recall, Benson Complex Figure Copy Delayed Recall, MiNT, Letter Fluency (L & C).

Global cognitive, functional, mood and gait speed assessments:

Clinical Dementia Rating (CDR): measures dementia severity and is a global rating of dementia with scores ranging from 0 to 3 (0, 0.5, 1, 2, and 3) rated by a semi-structured subject and informant interview[83]. A clinician synthesizes the cognitive and functional abilities based on 6 domains, including memory, orientation, judgment and problem solving, community affairs, home and hobbies, and personal care. The scale has good inter-rater agreement[83]. Participants with a CDR score < 1.0 will be eligible and confirmed clinically as non-demented.

MMSE: The Folstein MMSE is a 30-point scale is a global cognitive measure collected to facilitate comparison across studies and used to determine eligibility. Potential participants with MMSE >23 will be eligible[84].

Montreal Cognitive Assessment (MoCA): A rapid screening instrument for assessing different cognitive domains. Subjects are asked to do the following tasks: 1.) Join 10 randomly displayed numbers and letters in an alternating and sequential fashion. 2.) To measure visuoconstructional skills, accurately copy two drawings, a cube and a clock. 3.) Provide the names of three animals represented by drawings. 4.) Remember a list of 5 words. This is repeated 2 times. After a delay, subjects again recall as many words as possible. 5.) To

measure attention: repeat a five number sequence forward, and a separate three number sequence backwards, signal when a certain letter is read in a spoken sequence of varying letters, and count backwards from 100 by subtracting seven. 6.) Name as many words that start with the letter "F" in sixty seconds. 7.) Explain what pairs of words have in common, such as "Tell me how an orange and a banana are alike." 8.) To provide orientation, provide date, place, and city. [Admin. 10 min.]

Geriatric Depression Scale-15. The GDS-15 is a widely used instrument for screening depression in the elderly. It is brief, non-somatically focused, and can be either observer or self-administered. The scores range from 0 to 15, with higher scores indicating more severe depression. The GDS-15 was developed and originally validated in elderly patients[85]. [Admin 5 mins]

Instrumental Activities of Daily Living: Measures an individual's ability to carry out tasks that are important for daily living. The IADLs are collected to capture functional changes[90] [Admin 10 minutes]

Timed Gait Test: WMH is associated with slowed gait speed[8, 19, 86-88]. A 10-yard (30 foot) course will be measured and marked on a straight unobstructed area prior to test administration. Participants will be instructed to walk the 10 yards as quickly as possible without running, cross a line, turn around, and walk back the 10 yards. The average time of three trials is recorded in seconds.[89]

Central (Aortic) Blood Pressure
CardioScope TM is a non-invasive, compact standalone measurement device that automatically measures systolic and diastolic pressure, and pulse rate in adult and pediatric patients. CardioScope also provides non-invasive central (aortic) systolic, mean and diastolic blood pressure, and pulse waveform intended for use in adult patients. The CardioScope first measures brachial systolic and diastolic blood pressures and pulse rate using the Oscillometric method. It then inflates the cuff to a pressure about 30 mmHg higher than systolic (Suprasystolic) pressure to acquire a further oscillometric pulse waveform. The suprasystolic pulse waveform is used in combination with the conventional upper-arm blood pressures to derive central systolic, diastolic and mean blood pressures. Central blood pressure will be taken at Screen II (visit 2), and months 12, 24, and 36.

Safety labs. Will include a CBC, CMP and PT/INR. These labs are assessed at screen, 6, 12, 18, 24, and 36 months.

Year		1	2	3	4	5
**IRB approval**		X				
**Enrollment at Baseline**		N=75	N=150	Attrition n=15	Attrition n=14	Attrition n=11, n=110
**Intervention**		X	X	X	X	X
**Data collection**		X	X	X	X	X
**Data Analysis**		Enrollment stats	Enrollment stats	Baseline stats		Primary outcomes

E. HUMAN SUBJECTS
1. Risks To Human Subjects
<u>1.A. Human subjects involvement, Characteristics and Design</u>
A total of 150 participants age 75 and older will be enrolled, and randomized to assigned treatment group after meeting screening criteria; will be followed over three years. Participants will be recruited from Oregon Health & Science University, Portland, OR and the surrounding communities including Vancouver, Washington. Participants will non-demented and have a CDR of ≤ 0.5, and screening will include a MRI and blood test to enrich the population with white matter lesions and relatively lower plasma pufas. The Inclusion/Exclusion criteria are described under Approach, Study Population. We will recruit approximately equal numbers of men and women, and no ethnic group will be excluded. Because of the demographics in the state of Oregon we do expect to recruit a larger number of white non-Hispanic than might be reflected across the U.S. Eighty-seven percent of all Oregonians are white with about 10% combined being Black, Asian or Pacific Islander, or Hispanic. Study inclusion requires all study participants have a partner that spends at least 1 hour/day or 1 day per week with the participant to corroborate independence of activities of daily living. Non-demented

persons of advanced age are particularly vulnerable to brain health changes, which are insidious in younger populations. Enriching our study population by advanced age, white matter disease and low pufas status is necessary to assure higher power to test our hypothesis that pufas slow white matter disease and the associated cognitive consequences of its progression. Allocation of study supplement assignment is double blinded and randomized 50:50. The intervention is chosen on the basis of our first-hand experience and proven safety and efficacy in raising the key pufas to a predictable tissue level.

1.B. Sources of materials

Study measures will be collected from participants ten times over the 36-month intervention (screen/baseline, 3, 6, 12, 18, 24, 30, and 36-months). Study measures include brain MRI, Cognitive, Global, Functional and Behavioral assessments, blood and DNA. Outcomes include, MRI, Cognitive (attention and processing speed, working memory, executive function, memory, language, visuospatial skills and global cognition), Functional (ADLs, gait speed), blood pressure,, blood-based biomarkers of endothelial function and lipid metabolism collected at baseline, 3 and 6 months, and then every 6 months over 3 years. Central aortic blood pressure will be collected at Screening Visit II, and then once every year. Safety lab work (CBC, CMP, and PT/INR. Data from safety labs measured at screening, 6, 12, 18, 24 and 36 months will be processed and reviewed for safety. Medical record reports, including labs and MRI report along with PE/neurological exam, will be used to help with inclusion/exclusion criteria on the screening visits. Once the treatment intervention begins subject AE will be collected in “real time” and discussed during weekly team meeting. Data for compliance by pill count will be collected every 3 months. Drs. Hiroko Dodge (statistician), Lynne Shinto (site PI), and Gene Bowman (grant PI) along with the Charles Murchison, informatics core of OHSU’s NeuroNext will facilitate data collection, management and protection. Dr. Hiroko Dodge will perform the data analysis. All data sources will have a unique ID number that has no personal identifiable information to support confidentiality. All study files will be stored in locked file cabinets and keys provided under the supervision of the Dr. Lynne Shinto (site PI) for appropriate staff (e.g. study coordinator, research assistant). All data obtained from this study will be used for research purposes only and will comply with HIPAA regulations.

1.C. Potential Risks

Venipuncture: The risks are minimal and may include some pain, bleeding and transient hematomas.
Questionnaires: The risk from filling out diet habit questionnaires and psychometric tests are minimal, there may be mild anxiety from filling out questionnaires.
Omega-3 pufas and soybean oil placebo: At the daily dose given in this study, 1650 mg of omega 3 pufas (975 mg EPA + 675 mg DHA), the risks are minimal. This dose is Generally Regarded As Safe (GRAS) by the FDA. Potential side effects include fishy belches, and mild gastrointestinal effects (nausea, loose stools, indigestion). Because fish oil does have anti-platelet and anti-thromboxane A 2 effects there is a concern about increased bleeding time and interactions with blood thinning medications (e.g. warfarin). Although a study evaluating the effects of 1-year of fish oil supplementation on patients undergoing artery bypass reported no increase in bleeding during surgery in patients given fish oil plus aspirin or given fish oil plus warfarin compared to patients given those therapies alone. This study will monitor bleeding time by PT/INR as a protection against this risk. Soybean oil will be given at 3 grams per day, like the omega 3 pufa; the risks are minimal and may include mild gastrointestinal effects.
As a protection against any risk, adverse event reports will be monitored every 3 months either by phone or in-clinic and participants. A CBC, comprehensive metabolic panel will be collected at screening, 6, 12, 18, 24 and 36 months to monitor any treatment effects on organ function as another protection against risk.
MRI: Risk to having a brain MRI is minimal. Feeling claustrophobic is a risk. We are excluding subjects with MRI contraindication (i.e. pacemakers, cranial plates) to minimize risks.

2. Adequacy Of Protection Against Risks

2.A. Recruitment and Informed Consent

Recruitment: Prior to recruitment, the OHSU IRB will approve this study for human study. The study will recruit from the NIA-Layton Aging and Alzheimer’s Disease Center at OHSU, which serves the entire state of Oregon. The study will be advertised in local retirement communities, newspapers, radio, television, and the NIH Clinical Trials website with associated press release. A blinded scan of the OHSU clinical database determines how many potential participants have visited OHSU in the last year. A recent scan that included people meeting our inclusion criteria, including: age 80 and older who have visited the academic medical center within the past year, without a diagnosis of a dementing illness, free of contraindications for MRI

(pacemaker), without ventricular-tachycardia, ventricular fibrillation, IDDM, cancer and residence within close proximity to OHSU (Oregon and Washington counties close to Portland and Bend), Oregon (counties include Multnomah, Clark, Clackamas, Washington, Columbia, Polk, Marion, Deschutes) identified 6,571 eligible men and women for recruitment. Assuming 20% of this population has no evidence of WMH and plasma pufas too high then that leaves 5,256 elders. We would need to recruit <3% of this population to satisfy our recruitment goals. **Individuals identified through medical record scans will be sent an IRB approved letter describing their potential eligibility for the study. We will also utilize NeuroNext database of people that have consented to be contacted about clinical trial opportunities at OHSU.** In addition, the NIA-Layton Aging & Alzheimer's Disease Center maintains direct contact for its ongoing longitudinal aging studies in over 50 aging centers, retirement communities and community organizations in the Portland metro area. To compensate for cost of travel, time and effort each participant will receive $40/complete visit to OHSU. **Individuals who complete a combined visit 1 and 2 will receive $80 for their first visit and $40 for any subsequent visits.** Participants traveling to OHSU for MRI scan from the > 100 miles outside of Portland will be compensated at $200 for each visit to OHSU for gas and overnight lodging costs.

Informed Consent: Prior to the in-clinic screening visit, all potential participants and their study partners will be mailed a copy of the IRB approved consent/HIPAA authorization form so that they may be fully informed of all aspects of their participation in the study. At the time of consent one of the study investigators, coordinators, or the research assistants will review the consent/HIPAA authorization form and then will ask the participant and study partner to describe key points in the consent form (e.g. purpose of study, knowledge of placebo control group, length of study) before the consent form will be signed. Once the participant signs the consent form he or she will continue with screening assessments.

2.B. Protection Against Risk

All information about subjects will be locked so that only authorized study staff will have access. Research records will be coded with a unique patient identification number; therefore will contain no personal identifiers. All records with identifying information will be locked so that only authorized study staff will have access to them. Data will be kept in locked cabinets separate from identifiers. Computer files will be password protected. No one other than the researchers will have access to identifiable data. Participants will be told that they have the right to refuse to answer any question(s). If they are upset by a question they will be referred to the Principal Investigator. We will monitor adverse events by laboratory tests, which include prothrombin time (PT/INR), CBC and CMP and by every 3-month adverse events report. We are monitoring the effects of treatment on CBC and CMP at baseline, 3, 6, 12, 18, 24 and 36-months. Subjects whose labs reflect significant abnormalities, at 3 months (that have not reported adverse events) will be further screened to determine the potential cause(s) of the lab abnormalities; if the study treatments are suspected, we will discontinue treatment and remove the subject from the study. A data safety monitoring board (DSMB) will convene biannually to study unblinded data on AE coded by organ system. All subjects will be given a telephone number and be instructed to call Dr. Shinto or the trial coordinator if they believe they are experiencing severe side effects from the study medication.

Plan for informing participants and their caretakers of incidental findings with potential significance from MRI: The MRI derived from this study will be reviewed for clinically relevant findings that would impact health (i.e., cortical stroke, tumor, normal pressure hydrocephalus). If clinically relevant findings are identified, Dr. Silbert or Dr. Quinn will contact a neuroradiologist for a clinical read within 78 hours of identified finding.. The PI or study coordinator will notify the subject by phone within one week of the discovery to discuss the abnormal finding. The subject's PCP will be notified of the finding and the clinical brain scan will be provided to support subject the appropriate care.. Dr. Shinto (sitePI) will also work with the PCP to facilitate an expedited referral to a specialist (e.g., neurologist, neuroradiologist) if the PCP deems necessary.

3. Potential Benefits Of The Proposed Research To The Subjects And Others

PUFAs have demonstrated anti-inflammatory, and more specifically, anti-leukocyte migration properties. They may have potential benefit for chronic disorders that have an inflammatory component. PUFAs have also been reported to decrease triglyceride levels, blood pressure and reduce cardiac related mortality so this therapy may be beneficial to subjects that are at risk for vascular disease.

4. Importance Of Knowledge To Be Gained

This study will test the safety and effectiveness of pufas in favorably modifying vascular contributions to cognitive decline in non-demented elders. Because there are no current therapies that prevent age-related cognitive decline, it is an important public health issue to scientifically evaluate this therapy and learn the potential for risk reduction.

5. Data And Safety Monitoring Plan

Adverse events will be monitored in "real time" with weekly review of AEs with study staff using clinical judgment to determine reporting to IRB and FDA. A data safety monitoring board (DSMB) will convene biannually to study unblinded data on AE coded by organ system. Although assessment of AEs will also include solicitation of non-specific change (e.g., "do you feel different since beginning the treatment?"), a 12-item AE checklist covering all major organ systems will be included to probe for AEs. The nature of each AE, its severity (mild, moderate, or severe), its likely relationship to study treatment (definite, probable, possible, not related, or unknown), its duration, and any necessary treatment modifications or adjustments will be recorded. In addition to the monitoring and recording of AEs, labs to assess basic metabolic function (including liver function tests), CBC and prothrombin time will be performed at screening and months 6, 12, 18, 24 and 36. Participants will be reminded and encouraged during clinic visits and phone checks to contact the study coordinator or investigator if a moderate or serious AE occurs.

References

1. Virtanen JK, Siscovick DS, Longstreth WT, Jr., Kuller LH, Mozaffarian D. Fish consumption and risk of subclinical brain abnormalities on MRI in older adults. Neurology 2008;71:439-446.
2. Bowman GL, Silbert LC, Howieson D, et al. Nutrient biomarker patterns, cognitive function, and MRI measures of brain aging. Neurology 2012;78:241-249.
3. Gorelick PB, Scuteri A, Black SE, et al. Vascular Contributions to Cognitive Impairment and Dementia: A Statement for Healthcare Professionals From the American Heart Association/American Stroke Association. Stroke 2011.
4. Unverzagt FW, McClure LA, Wadley VG, et al. Vascular risk factors and cognitive impairment in a stroke-free cohort. Neurology 2011;77:1729-1736.
5. Barnes DE, Yaffe K. The projected effect of risk factor reduction on Alzheimer's disease prevalence. Lancet Neurol 2011;10:819-828.
6. Dodge HH, Chang CC, Kamboh IM, Ganguli M. Risk of Alzheimer's disease incidence attributable to vascular disease in the population. Alzheimers Dement 2011;7:356-360.
7. DeKosky ST, Williamson JD, Fitzpatrick AL, et al. Ginkgo biloba for prevention of dementia: a randomized controlled trial. Jama 2008;300:2253-2262.
8. Silbert LC, Nelson C, Howieson DB, Moore MM, Kaye JA. Impact of white matter hyperintensity volume progression on rate of cognitive and motor decline. Neurology 2008;71:108-113.
9. Silbert LC, Howieson DB, Dodge H, Kaye JA. Cognitive impairment risk: white matter hyperintensity progression matters. Neurology 2009;73:120-125.
10. Dufouil C, Chalmers J, Coskun O, et al. Effects of blood pressure lowering on cerebral white matter hyperintensities in patients with stroke: the PROGRESS (Perindopril Protection Against Recurrent Stroke Study) Magnetic Resonance Imaging Substudy. Circulation 2005;112:1644-1650.
11. Yue NC, Arnold AM, Longstreth WT, Jr., et al. Sulcal, ventricular, and white matter changes at MR imaging in the aging brain: data from the cardiovascular health study. Radiology 1997;202:33-39.
12. de Leeuw FE, de Groot JC, Achten E, et al. Prevalence of cerebral white matter lesions in elderly people: a population based magnetic resonance imaging study. The Rotterdam Scan Study. J Neurol Neurosurg Psychiatry 2001;70:9-14.
13. Baum KA, Schulte C, Girke W, Reischies FM, Felix R. Incidental white-matter foci on MRI in "healthy" subjects: evidence of subtle cognitive dysfunction. Neuroradiology 1996;38:755-760.
14. DeCarli C, Murphy DG, Tranh M, et al. The effect of white matter hyperintensity volume on brain structure, cognitive performance, and cerebral metabolism of glucose in 51 healthy adults. Neurology 1995;45:2077-2084.

15. Jokinen H, Ryberg C, Kalska H, et al. Corpus callosum atrophy is associated with mental slowing and executive deficits in subjects with age-related white matter hyperintensities: the LADIS Study. J Neurol Neurosurg Psychiatry 2007;78:491-496.
16. Longstreth WT, Jr., Manolio TA, Arnold A, et al. Clinical correlates of white matter findings on cranial magnetic resonance imaging of 3301 elderly people. The Cardiovascular Health Study. Stroke 1996;27:1274-1282.
17. Prins ND, van Dijk EJ, den Heijer T, et al. Cerebral white matter lesions and the risk of dementia. Arch Neurol 2004;61:1531-1534.
18. Schmidt R, Fazekas F, Offenbacher H, et al. Neuropsychologic correlates of MRI white matter hyperintensities: a study of 150 normal volunteers. Neurology 1993;43:2490-2494.
19. Starr JM, Leaper SA, Murray AD, et al. Brain white matter lesions detected by magnetic resonance [correction of resosnance] imaging are associated with balance and gait speed. J Neurol Neurosurg Psychiatry 2003;74:94-98.
20. Taylor MD, Hart CL, Davey Smith G, et al. Childhood mental ability and smoking cessation in adulthood: prospective observational study linking the Scottish Mental Survey 1932 and the Midspan studies. J Epidemiol Community Health 2003;57:464-465.
21. Sachdev P, Wen W, Chen X, Brodaty H. Progression of white matter hyperintensities in elderly individuals over 3 years. Neurology 2007;68:214-222.
22. Schmidt R, Enzinger C, Ropele S, Schmidt H, Fazekas F. Progression of cerebral white matter lesions: 6-year results of the Austrian Stroke Prevention Study. Lancet 2003;361:2046-2048.
23. Bastos Leite AJ, van der Flier WM, van Straaten EC, Scheltens P, Barkhof F. Infratentorial abnormalities in vascular dementia. Stroke 2006;37:105-110.
24. Garde E, Lykke Mortensen E, Rostrup E, Paulson OB. Decline in intelligence is associated with progression in white matter hyperintensity volume. J Neurol Neurosurg Psychiatry 2005;76:1289-1291.
25. Schmidt R, Schmidt H, Kapeller P, Fazekas F. Slow progression of white-matter changes. Int Psychogeriatr 2003;15 Suppl 1:173-176.
26. Kramer JH, Mungas D, Reed BR, et al. Longitudinal MRI and cognitive change in healthy elderly. Neuropsychology 2007;21:412-418.
27. Debette S, Beiser A, DeCarli C, et al. Association of MRI markers of vascular brain injury with incident stroke, mild cognitive impairment, dementia, and mortality: the Framingham Offspring Study. Stroke 2010;41:600-606.
28. Adak S, Illouz K, Gorman W, et al. Predicting the rate of cognitive decline in aging and early Alzheimer disease. Neurology 2004;63:108-114.
29. Brickman AM, Zimmerman ME, Paul RH, et al. Regional white matter and neuropsychological functioning across the adult lifespan. Biol Psychiatry 2006;60:444-453.
30. Verdelho A, Madureira S, Ferro JM, et al. Differential impact of cerebral white matter changes, diabetes, hypertension and stroke on cognitive performance among non-disabled elderly. The LADIS study. J Neurol Neurosurg Psychiatry 2007;78:1325-1330.
31. Bowman GL, Kaye JA, Quinn JF. Dyslipidemia and blood-brain barrier integrity in Alzheimer's disease. Curr Gerontol Geriatr Res 2012;2012:184042.
32. Lloyd-Jones DM, Hong Y, Labarthe D, et al. Defining and setting national goals for cardiovascular health promotion and disease reduction: the American Heart Association's strategic Impact Goal through 2020 and beyond. Circulation 2010;121:586-613.
33. Freund-Levi Y, Eriksdotter-Jonhagen M, Cederholm T, et al. Omega-3 fatty acid treatment in 174 patients with mild to moderate Alzheimer disease: OmegAD study: a randomized double-blind trial. Arch Neurol 2006;63:1402-1408.
34. Quinn JF, Raman R, Thomas RG, et al. Docosahexaenoic acid supplementation and cognitive decline in Alzheimer disease: a randomized trial. Jama 2010;304:1903-1911.
35. Huang TL, Zandi PP, Tucker KL, et al. Benefits of fatty fish on dementia risk are stronger for those without APOE epsilon4. Neurology 2005;65:1409-1414.
36. Cawood AL, Ding R, Napper FL, et al. Eicosapentaenoic acid (EPA) from highly concentrated n-3 fatty acid ethyl esters is incorporated into advanced atherosclerotic plaques and higher plaque EPA is associated with decreased plaque inflammation and increased stability. Atherosclerosis 2010;212:252-259.
37. Bouwens M, van de Rest O, Dellschaft N, et al. Fish-oil supplementation induces antiinflammatory gene expression profiles in human blood mononuclear cells. Am J Clin Nutr 2009;90:415-424.

38. Shinto L, Marracci G, Baldauf-Wagner S, et al. Omega-3 fatty acid supplementation decreases matrix metalloproteinase-9 production in relapsing-remitting multiple sclerosis. Prostaglandins Leukot Essent Fatty Acids 2009;80:131-136.
39. Hogg N, Bates PA, Harvey J. Structure and function of intercellular adhesion molecule-1. Chem Immunol 1991;50:98-115.
40. Witkowska AM, Borawska MH. Soluble intercellular adhesion molecule-1 (sICAM-1): an overview. Eur Cytokine Netw 2004;15:91-98.
41. Jun CD, Shimaoka M, Carman CV, Takagi J, Springer TA. Dimerization and the effectiveness of ICAM-1 in mediating LFA-1-dependent adhesion. Proc Natl Acad Sci U S A 2001;98:6830-6835.
42. Jun CD, Carman CV, Redick SD, Shimaoka M, Erickson HP, Springer TA. Ultrastructure and function of dimeric, soluble intercellular adhesion molecule-1 (ICAM-1). J Biol Chem 2001;276:29019-29027.
43. Dietrich JB. The adhesion molecule ICAM-1 and its regulation in relation with the blood-brain barrier. J Neuroimmunol 2002;128:58-68.
44. Camacho SA, Heath WR, Carbone FR, et al. A key role for ICAM-1 in generating effector cells mediating inflammatory responses. Nat Immunol 2001;2:523-529.
45. Debette S, Bis JC, Fornage M, et al. Genome-wide association studies of MRI-defined brain infarcts: meta-analysis from the CHARGE Consortium. Stroke 2010;41:210-217.
46. Hassan A, Hunt BJ, O'Sullivan M, et al. Markers of endothelial dysfunction in lacunar infarction and ischaemic leukoaraiosis. Brain 2003;126:424-432.
47. Markus HS, Hunt B, Palmer K, Enzinger C, Schmidt H, Schmidt R. Markers of endothelial and hemostatic activation and progression of cerebral white matter hyperintensities: longitudinal results of the Austrian Stroke Prevention Study. Stroke 2005;36:1410-1414.
48. Han JH, Wong KS, Wang YY, Fu JH, Ding D, Hong Z. Plasma level of sICAM-1 is associated with the extent of white matter lesion among asymptomatic elderly subjects. Clin Neurol Neurosurg 2009;111:847-851.
49. de Leeuw FE, de Kleine M, Frijns CJ, Fijnheer R, van Gijn J, Kappelle LJ. Endothelial cell activation is associated with cerebral white matter lesions in patients with cerebrovascular disease. Ann N Y Acad Sci 2002;977:306-314.
50. Yang Y, Lu N, Chen D, Meng L, Zheng Y, Hui R. Effects of n-3 PUFA supplementation on plasma soluble adhesion molecules: a meta-analysis of randomized controlled trials. Am J Clin Nutr 2012;95:972-980.
51. Corrada MM, Brookmeyer R, Berlau D, Paganini-Hill A, Kawas CH. Prevalence of dementia after age 90: results from the 90+ study. Neurology 2008;71:337-343.
52. Jellinger KA, Attems J. Prevalence of dementia disorders in the oldest-old: an autopsy study. Acta Neuropathol 2010;119:421-433.
53. Middleton LE, Grinberg LT, Miller B, Kawas C, Yaffe K. Neuropathologic features associated with Alzheimer disease diagnosis: age matters. Neurology 2011;77:1737-1744.
54. He K, Rimm EB, Merchant A, et al. Fish consumption and risk of stroke in men. Jama 2002;288:3130-3136.
55. Iso H, Rexrode KM, Stampfer MJ, et al. Intake of fish and omega-3 fatty acids and risk of stroke in women. Jama 2001;285:304-312.
56. Mozaffarian D, Longstreth WT, Jr., Lemaitre RN, et al. Fish consumption and stroke risk in elderly individuals: the cardiovascular health study. Arch Intern Med 2005;165:200-206.
57. Leaf A, Albert CM, Josephson M, et al. Prevention of fatal arrhythmias in high-risk subjects by fish oil n-3 fatty acid intake. Circulation 2005;112:2762-2768.
58. Mozaffarian D, Bryson CL, Lemaitre RN, Burke GL, Siscovick DS. Fish intake and risk of incident heart failure. J Am Coll Cardiol 2005;45:2015-2021.
59. Rapp SR, Espeland MA, Shumaker SA, et al. Effect of estrogen plus progestin on global cognitive function in postmenopausal women: the Women's Health Initiative Memory Study: a randomized controlled trial. Jama 2003;289:2663-2672.
60. Martin BK, Szekely C, Brandt J, et al. Cognitive function over time in the Alzheimer's Disease Anti-inflammatory Prevention Trial (ADAPT): results of a randomized, controlled trial of naproxen and celecoxib. Arch Neurol 2008;65:896-905.
61. van de Rest O, Geleijnse JM, Kok FJ, et al. Effect of fish oil on cognitive performance in older subjects: a randomized, controlled trial. Neurology 2008;71:430-438.
62. Dangour AD, Allen E, Elbourne D, et al. Effect of 2-y n-3 long-chain polyunsaturated fatty acid supplementation on cognitive function in older people: a randomized, double-blind, controlled trial. Am J Clin Nutr 2010;91:1725-1732.

63. Geleijnse JM, Giltay EJ, Kromhout D. Effects of n-3 fatty acids on cognitive decline: A randomized, double-blind, placebo-controlled trial in stable myocardial infarction patients. Alzheimers Dement 2012;8:278-287.
64. Cole GM, Ma QL, Frautschy SA. Omega-3 fatty acids and dementia. Prostaglandins Leukot Essent Fatty Acids 2009;81:213-221.
65. Tanaka K, Ishikawa Y, Yokoyama M, et al. Reduction in the recurrence of stroke by eicosapentaenoic acid for hypercholesterolemic patients: subanalysis of the JELIS trial. Stroke 2008;39:2052-2058.
66. Brickman AM, Zimmerman ME, Paul RH, et al. Regional white matter and neuropsychological functioning across the adult lifespan. Biological psychiatry 2006;60:444-453.
67. Verdelho A, Madureira S, Ferro JM, et al. Differential impact of cerebral white matter changes, diabetes, hypertension and stroke on cognitive performance among non-disabled elderly. The LADIS study. Journal of neurology, neurosurgery, and psychiatry 2007;78:1325-1330.
68. Schouten HJ. Adaptive biased urn randomization in small strata when blinding is impossible. Biometrics 1995;51:1529-1535.
69. Little RJAaR, D.B. Statistical Analysis with Missing Data, 2nd ed. New York: John Wiley, 2002.
70. Jack CR, Jr., Bernstein MA, Fox NC, et al. The Alzheimer's Disease Neuroimaging Initiative (ADNI): MRI methods. J Magn Reson Imaging 2008;27:685-691.
71. Fazekas F, Chawluk JB, Alavi A, Hurtig HI, Zimmerman RA. MR signal abnormalities at 1.5 T in Alzheimer's dementia and normal aging. AJR Am J Roentgenol 1987;149:351-356.
72. Smith SM, Jenkinson M, Woolrich MW, et al. Advances in functional and structural MR image analysis and implementation as FSL. Neuroimage 2004;23 Suppl 1:S208-219.
73. Brickman AM, Sneed JR, Provenzano FA, et al. Quantitative approaches for assessment of white matter hyperintensities in elderly populations. Psychiatry Res 2011;193:101-106.
74. Samieri C, Maillard P, Crivello F, et al. Plasma long-chain omega-3 fatty acids and atrophy of the medial temporal lobe. Neurology 2012.
75. Jovicich J, Czanner S, Han X, et al. MRI-derived measurements of human subcortical, ventricular and intracranial brain volumes: Reliability effects of scan sessions, acquisition sequences, data analyses, scanner upgrade, scanner vendors and field strengths. Neuroimage 2009;46:177-192.
76. Desikan RS, Cabral HJ, Fischl B, et al. Temporoparietal MR imaging measures of atrophy in subjects with mild cognitive impairment that predict subsequent diagnosis of Alzheimer disease. AJNR Am J Neuroradiol 2009;30:532-538.
77. Witte V, Kerti, L., Agnes, N. Effects of omega 3 supplementation on brain structure and function in healthy elderly subjects. Alzheimer's Association International Conference Vancouver, B.C.2012.
78. Smith SM JM, Johansen-Berg H, Rueckert D, Nichols TE, Mackay CE, Watkins KE, Ciccarelli O, Cader MZ, Matthews PM, Behrens TE. Tract-based spatial statistics: voxelwise analysis of multi-subject diffusion data. Neuroimage 2006;31:1487-1505.
79. Smith SM, Zhang Y, Jenkinson M, et al. Accurate, robust, and automated longitudinal and cross-sectional brain change analysis. Neuroimage 2002;17:479-489.
80. Vartanian K, Slottke R, Johnstone T, et al. Gene expression profiling of whole blood: comparison of target preparation methods for accurate and reproducible microarray analysis. BMC genomics 2009;10:2.
81. Bowman GL, Shannon J, Ho E, et al. Reliability and validity of food frequency questionnaire and nutrient biomarkers in elders with and without mild cognitive impairment. Alzheimer Dis Assoc Disord 2011;25:49-57.
82. Gaudino EA, Geisler MW, Squires NK. Construct validity in the Trail Making Test: what makes Part B harder? J Clin Exp Neuropsychol 1995;17:529-535.
83. Salthouse TA. What do adult age differences in the Digit Symbol Substitution Test reflect? J Gerontol 1992;47:P121-128.
84. Dodge KA. Framing public policy and prevention of chronic violence in American youths. Am Psychol 2008;63:573-590.
85. Folstein MF, Folstein SE, McHugh PR. "Mini-mental state". A practical method for grading the cognitive state of patients for the clinician. J Psychiatr Res 1975;12:189-198.
86. Yesavage JA. Geriatric Depression Scale. Psychopharmacol Bull 1988;24:709-711.
87. Baezner H, Blahak C, Poggesi A, et al. Association of gait and balance disorders with age-related white matter changes: the LADIS study. Neurology 2008;70:935-942.
88. Camicioli R, Moore MM, Sexton G, Howieson DB, Kaye JA. Age-related brain changes associated with motor function in healthy older people. J Am Geriatr Soc 1999;47:330-334.

89. Rosano C, Brach J, Longstreth Jr WT, Newman AB. Quantitative measures of gait characteristics indicate prevalence of underlying subclinical structural brain abnormalities in high-functioning older adults. Neuroepidemiology 2006;26:52-60.
90. Robertson KR, Parsons TD, Sidtis JJ, et al. Timed Gait test: normative data for the assessment of the AIDS dementia complex. J Clin Exp Neuropsychol 2006;28:1053-1064.
91. Davis SW, Potok MA, Brinkmeier ML, et al. Genetics, gene expression and bioinformatics of the pituitary gland. Horm Res 2009;71 Suppl 2:101-115.
92. Bligh EG, Dyer WJ. A rapid method of total lipid extraction and purification. Can J Biochem Physiol, 1959. 37(8): p. 911-7.
93. Johnston, D.T. et al. Red blood cell omega-3 fatty acid levels and neurocognitive performance in deployed U.S. servicemembers. Nutri. Neurosci., 2012. 16(1): p. 30-38.
