# Supplementary material for: ω-3 PUFA for Secondary Prevention of White Matter Lesions and Neuronal Integrity Breakdown in Older Adults: A Randomized Clinical Trial
Source: JAMA Netw Open. 2024 Aug 1;7(8):e2426872. doi: 10.1001/jamanetworkopen.2024.26872 (PMC11294966; doi:10.1001/jamanetworkopen.2024.26872)
Supplement: Supplement 2. — eTable 1. PUFA trial eligibility criteria eTable 2. Independent laboratory testing of ω-3 PUFA and soybean oil placebo illustrating a potent (total mg/mL oil/capsule and % concentration of label claim) and stabile (lipid oxidationa, b, c) supplement over trial duration eAppendix. Supplemental methods eTable 3. ω-3 PUFA effects on exploratory outcomes and by apoE genotype over 3-yearsa eReferences [file jamanetwopen-e2426872-s002.pdf]

Supplemental Online Content

Shinto LH, Murchison CF, Silbert LC, et al. ω-3 PUFA for secondary prevention of white matter lesions and neuronal integrity breakdown in older adults: a randomized clinical trial. *JAMA Netw Open*. 2024;7(8):e2426872.
doi:10.1001/jamanetworkopen.2024.26872

eTable 1. PUFA trial eligibility criteria
eTable 2. Independent laboratory testing of ω-3 PUFA and soybean oil placebo illustrating a potent (total mg/mL oil/capsule and % concentration of label claim) and stabile (lipid oxidation[a, b, c]) supplement over trial duration
eAppendix. Supplemental methods
eTable 3. ω-3 PUFA effects on exploratory outcomes and by apoE genotype over 3-years[a]
eReferences

This supplemental material has been provided by the authors to give readers additional information about their work.

© 2024 Shinto LH et al. *JAMA Network Open.*

eTable 1. PUFA trial eligibility criteria

Inclusion criteria

1. Non-demented or mild cognitive impairment, defined as Clinical Dementia Rating 0/0.5 and MMSE >23
2. Age 75 and older, male and female
3. Total WML volume $\geq 5\ cm^3$
4. Plasma ω-3 < 110 ug/ml or 5.5% in whole bloodspot
5. Sufficient English language skills to complete all tests
6. Geriatric Depression Scale - 15 below 6
7. Sufficient vision and hearing to complete all tests
8. Informant available with frequent (at least 1 hour/day or one day/week) contact with the subject to verify the functional status and CDR rating
9. General health status that will not interfere with the ability to complete the prospective study (these conditions are listed below in the study exclusion list)

Exclusion criteria

1. Any dementing illness (AD, vascular dementia, normal pressure hydrocephalus, or Parkinson's disease); dementia defined by CDR ≥ 1, MMSE < 24
2. A significant disease of the CNS such as brain tumor, seizure disorder, subdural hematoma, cranial arteritis
3. Alcohol or substance abuse according to DSM-IV criteria within the last two years
4. Major depression, schizophrenia, or other major psychiatric disorder defined by DSM-IV criteria
5. Abnormal labs indicating vitamin B12 deficiency, thyroid disease, or UTI (documented bacterial colonization is acceptable)
6. Unstable or significantly symptomatic CVD (e.g., CAD with frequent angina, CHF with dyspnea at rest)
7. Hypertension as uncontrolled BP above 150/90
8. Clinical symptomatic orthostatic hypotension
9. Diabetes mellitus that requires insulin injections
10. History of cortical infarct

© 2024 Shinto LH et al. *JAMA Network Open.*

11. Cancer within the last five years, except localized prostate cancer (Gleason Grade < 3) and non-metastatic skin cancers (melanoma).
12. An illness that requires >1 visit /month to a clinician
13. Contraindications to MRI (i.e., heart pacemaker, metal plates or objects in the head, claustrophobia)
14. Medications and supplementation[1]

---

[1]CNS active meds that have not been on stable doses for at least two months (cimetidine, beta-blockers, and SSRIs); Neuroleptics, antiparkinsonian agents, systemic corticosteroids, and narcotic analgesics; in the case where these were used for a self-limited time, they must have been discounted for a period of five half-lives before the baseline visit; Over the counter supplements are not by themselves exclusionary; however, subjects are asked not to change the dosing regimen throughout the trial unless medically indicated; the presence and dose of these agents are recorded; Cholinesterase inhibitors (i.e., Aricept); Investigational drugs within five half-lives before baseline; Anti-coagulation therapy: Vitamin K antagonist: warfarin (Coumadin, jantoven), Factor Xa inhibitors: rivaroxaban (xarelto), fondaparinux (arixtra), dibigatran (pradaxa), apixaban (eliquis); Low molecular weight heparins: dalteparin (fragmin), enoxaparin (lovenox)(Incident use of anticoagulant therapy will exclude further study drug allocation. However, participants will be asked to complete all follow-up study visits); A baseline screen plasma omega-3 above 5.5 weight percent of total whole blood EPA+DHA will confirm supplementation of omega-3 history. If an individual indicates regular supplementation with fish oil on the phone screen, can abstain four months before the study, visit one.

© 2024 Shinto LH et al. *JAMA Network Open.*

eTable 2: Independent laboratory testing of ω-3 PUFA and soybean oil placebo illustrating a potent (total mg/mL oil/capsule and % concentration of label claim) and stabile (lipid oxidation[a, b, c]) supplement over trial duration

Batch lot #	Analysis date	Arm	EPA 20:5ω -3, mg/ml	EPA 20:5ω-3 Percentage of 325 mg label claim	DHA 22:6ω -3, mg/ml	DHA 22:6ω-3 Percentage of 225 mg label claim	[a]Acid, KOH/g	[b]p-anisidine	[c]Peroxide, meq/kg	Oxidation, total
131724	03/19/15	Active	309.52	95.24	229.43	101.97	0.17	42.75	4.07	N/A
1302405	03/19/15	Placebo	1.41	0.43	0.97	0.43	0.23	33.13	5.94	N/A
150047	10/27/15	Active	320.37	98.58	225.86	100.38	1.91	42.76	5.22	53.20
1301313	10/27/15	Placebo	1.56	0.48	1.16	0.52	0.29	25.49	7.56	40.61
154591	09/27/16	Active	322.61	99.26	245.08	108.92	1.63	38.40	4.36	47.12
1401313	09/27/16	Placebo	1.71	0.53	1.16	0.52	0.28	19.24	13.85	46.94
155102	03/15/17	Active	329.70	101.45	218.41	97.07	0.12	37.83	6.53	50.89
1401313	03/15/17	Placebo	2.00	0.62	1.42	0.63	0.25	19.69	16.49	52.67
170241	09/14/17	Active	350.83	107.95	234.85	104.38	0.37	42.50	3.96	50.42
171735	09/14/17	Placebo	1.73	0.53	1.50	0.67	0.23	32.98	5.00	43.06
170241	03/08/18	Active	347.91	107.05	234.32	104.14	0.31	43.65	4.78	53.21
171735	03/08/18	Placebo	1.76	0.54	1.26	0.56	0.17	36.77	5.02	46.81
172984	09/12/18	Active	319.58	98.33	224.47	99.76	0.21	27.03	3.61	34.25
171735	09/12/18	Placebo	1.91	0.59	1.98	0.88	0.17	24.92	9.77	44.46
172984	03/14/19	Active	347.80	107.02	243.07	108.03	0.21	28.18	5.94	40.06
171735	03/14/19	Placebo	0.02	0.01	1.90	0.84	0.17	23.75	12.56	48.87

[a]Acid value is the milligrams of potassium hydroxide necessary to neutralize the free acids in 1 gm test sample;

[b]p-anisidine is defined by convention as 100 times the optical density measured at 350 nm in a 1-cm cuvette of a solution containing 1.0 gm of the oil in 100 mL of mixture of solvent and reagent. This value determines the quantity of aldehydes (principally 2-alkynals and 2,4-dienyls) in oil by reaction in an acetic acid solution of the aldehydic compounds in an oil and the p-anisidine, and then measuring absorbance at 350 nm;

[c]Peroxide in terms of milliequivalents of peroxide per 1000 gm of test sample that oxidize potassium iodide under conditions of the test. The substances are generally assumed to be peroxides or other similar products of fat oxidation.

© 2024 Shinto LH et al. *JAMA Network Open.*

eAppendix. Supplemental methods.

Neuropsychological and other functional assessments

The National Alzheimer's Coordinating Center Uniform Data Set Version 3 (UDS V3) neuropsychological battery was conducted with each participant, as previously described.[1] In addition to MMSE used for screening, the MoCA (Montreal Cognitive Assessment)[2] (an item in the UDS V3) and the Alzheimer's Disease Cooperative Study - Activities of Daily Living (ADCS iADL)[3] were collected at baseline and annually over 3-years.

Supplemental methods. Analytical approach to model fitness and potential confounding

Interactions between the adjusting covariates listed above and time were evaluated to assess confounding of the treatment effects but were found in all cases to be non-significant and were not retained in the final models with only main mean-effect terms included. Quadratic time variables were also considered but failed to improve model fitness, with linear terms used in the final model. Outcome skewness in WML and ventricle volume was remedied by log transformation before hypothesis testing to conform to the model assumptions. Model fitness was examined using formal fit criteria (i.e., leverage, model residuals, Cook's distance) and visual inspection of the residual plots verified the linear model assumptions. Sensitivity analysis evaluated at each time point by mixed model repeated measures (MMRM) with the within-subject error variance-covariance structure following a heterogeneous autoregressive structure. The modified intention to treat analysis (ITT) included all randomized participants with at least one follow-up MRI after baseline to calculate change (ITT cohort: n=87 of 102 randomized, n=45 of 51 in the n-3 group; n=42 of 51 in placebo).

© 2024 Shinto LH et al. *JAMA Network Open.*

eTable 3. ω-3 PUFA effects on exploratory outcomes and by apoE genotype over 3-years[a]

	ω-3	Placebo	Linear differences	ω-3 effect size	
Exploratory outcomes	*Annual change (95% CI)* [b]	*Annual change (95% CI)*[b]	*Coefficient estimate (95% CI)*[c]	*t-stat*[§] *(p-value)*	*Cohen's d* [d]
Periventricular WML, cm^3 (log)	0.08812 (0.04914, 0.1271)	0.1124 (0.07421, 0.1503)	-0.02424 (-0.06760, 0.01903)	-1.09 (0.28)	-0.25
ApoE*e*4 positive	0.04099 (-0.03875, 0.1207)	0.1108 (0.04450, 0.1757)	-0.06982 (-0.1592, 0.01672)	-1.57 (0.13)	-0.66
ApoE*e*4 negative	0.1070 (0.06252, 0.1515)	0.1154 (0.06922, 0.1608)	-0.008410 (-0.05672, 0.04049)	-0.34 (0.74)	-0.09
Subcortical WML, cm^3 (log)	0.02629 (-0.04019, 0.09277)	0.04021 (-0.02466, 0.1050)	-0.01392 (-0.08909, 0.06068)	-0.36 (0.72)	-0.08
ApoE*e*4 positive	0.05129 (-0.07029, 0.1729)	0.09711 (-0.006211, 0.1977)	-0.04582 (-0.1729, 0.08691)	-0.68 (0.51)	-0.27
ApoE*e*4 negative	0.01115 (-0.0688, 0.09111)	0.007609 (-0.07467, 0.08931)	0.003544 (-0.08705, 0.09264)	0.08 (0.94)	0.02
DTI-RD, mm^2/sec	3.143e-06 (1.449e-06, 4.836e-06)	4.894e-06 (3.280e-06, 6.500e-06)	-1.751e-06 (-3.506e-06, -3.214e-09)	-1.96 (0.06)	-0.68
ApoE*e*4 positive	4.126e-06 (2.222e-08, 8.23e-06)	7.004e-06 (4.026e-06, 9.942e-06)	-2.878e-06 (-6.900e-06, 9.284e-07)	-1.44 (0.19)	-1.11
ApoE*e*4 negative	2.707e-06 (7.699e-07, 4.644e-06)	3.686e-06 (1.795e-06, 5.568e-06)	-9.787e-07 (-2.859e-06, 8.942e-07)	-1.02 (0.32)	-0.41
DTI-MD, mm^2/sec	2.882e-06 (1.377e-06, 4.386e-06)	4.174e-06 (2.736e-06, 5.602e-06)	-1.292e-06 (-2.763e-06, 1.796e-07)	-1.73 (0.10)	-0.63
ApoE*e*4 positive	4.112e-06 (4.618e-07, 7.762e-06)	5.776e-06 (3.065e-06, 8.453e-06)	-1.665e-06 (-5.091e-06, 1.617e-06)	-0.98 (0.36)	-0.72
ApoE*e*4 negative	2.323e-06 (5.628e-07, 4.083e-06)	3.249e-06 (1.549e-06, 4.937e-06)	-9.266e-07 (-2.590e-06, 7.361e-07)	-1.09 (0.29)	-0.49
DTI-AD, mm^2/sec	2.303e-06 (3.128e-07, 4.294e-06)	2.587e-06 (6.017e-07, 4.545e-06)	-2.834e-07 (-2.269e-06, 1.716e-06)	-0.28 (0.78)	-0.03
ApoE*e*4 positive	4.489e-06 (7.329e-07, 8.246e-06)	3.381e-06 (3.763e-08, 6.645e-06)	1.108e-06 (-2.622e-06, 4.866e-06)	0.58 (0.56)	0.11

© 2024 Shinto LH et al. *JAMA Network Open.*

ApoE*e*4 negative	1.541e-06	2.227e-06	-6.861e-07	-0.57	
	(-8.340e-07, 3.916e-06)	(-2.247e-07, 4.63e-06)	(-3.034e-06, 1.698e-06)	(0.57)	-0.06
Executive *Z-scores*[e]	-0.07212	-0.03368	-0.03844	-0.95	-0.22
	(-0.1300, -0.01428)	(-0.08916, 0.02201)	(-0.1179, 0.04133)	(0.35)	
ApoE*e*4 positive	-0.02857	-0.06527	0.03670	0.42	
	(-0.1613, 0.1041)	(-0.1728, 0.04615)	(-0.1395, 0.2021)	(0.67)	0.11
ApoE*e*4 negative	-0.09387	-0.02153	-0.07234	-1.46	
	(-0.1620, -0.02578)	(-0.09238, 0.04873)	(-0.1689, 0.02597)	(0.15)	-0.40

WML, white matter lesions as total white matter hyperintensities; ApoEe4, apolipoprotein E e4 carriers; Non-carriers, non-carriers of the apoEe4 allele; DTI-RD, Diffusion tensor imaging of radial diffusivity; MD, mean diffusivity; AD, axial diffusivity

[a]ITT cohort includes all randomized participants with at least one follow-up MRI (n=87) and ApoE genotype available for stratified analysis (n=84, 4 missing apoE genotype). Twenty-six apoE4 carriers (n=15 placebo and n=11 active) and 58 were non-ApoE4 carriers (n=28 placebo; n=30 in active).

[b]Annualized changes calculated from primary mixed-effects models as time trajectories for treatment arms;

[c]Linear response difference taken as the treatment-time interaction coefficient from the primary mixed-effects models; [§]Active treatment effect sizes taken as the t-statistic from the primary mixed-effects model for the treatment-time interaction;

[d]Cohen's d calculated from the primary mixed-effects models using t-statistic and degrees of freedom according to each outcome; [#]Baseline differences derived from primary mixed-effects models as the test statistic for main-effect of treatment;

[e]Executive *Z* scores calculated as the mean of Trail Making Test Part B and WAIS-R Digit symbol *Z*-scores.

© 2024 Shinto LH et al. *JAMA Network Open.*

eReferences

1. Bowman GL, Silbert LC, Dodge HH, et al. Randomized Trial of Marine n-3 Polyunsaturated Fatty Acids for the Prevention of Cerebral Small Vessel Disease and Inflammation in Aging (PUFA Trial): Rationale, Design and Baseline Results. *Nutrients*. Mar 29 2019;11(4)doi:10.3390/nu11040735
2. Dong Y, Sharma VK, Chan BP, et al. The Montreal Cognitive Assessment (MoCA) is superior to the Mini-Mental State Examination (MMSE) for the detection of vascular cognitive impairment after acute stroke. *J Neurol Sci*. Dec 15 2010;299(1-2):15-8. doi:10.1016/j.jns.2010.08.051
3. Galasko D, Bennett D, Sano M, et al. An inventory to assess activities of daily living for clinical trials in Alzheimer's disease. The Alzheimer's Disease Cooperative Study. *Alzheimer Dis Assoc Disord*. 1997;11 Suppl 2:S33-9.

© 2024 Shinto LH et al. *JAMA Network Open.*
