# Supplementary material for: ω-3 PUFA for Secondary Prevention of White Matter Lesions and Neuronal Integrity Breakdown in Older Adults: A Randomized Clinical Trial
Source: JAMA Netw Open. 2024 Aug 1;7(8):e2426872. doi: 10.1001/jamanetworkopen.2024.26872 (PMC11294966; doi:10.1001/jamanetworkopen.2024.26872)
Supplement: Supplement 3. — Data Sharing Statement [file jamanetwopen-e2426872-s003.pdf]

# Data Sharing Statement

Shinto. ω-3 PUFA for Secondary Prevention of White Matter Lesions and Neuronal Integrity Breakdown in Older Adults. *JAMA Netw Open*. Published August 01, 2024. doi:10.1001/jamanetworkopen.2024.26872

**Data**
**Data available:** Yes
**Data types:** Deidentified participant data
**How to access data:** glbowman@mgh.harvard.edu or shintol@ohsu.edu
**When available:** 3 months after publication and up to 5 years following article publication

**Supporting Documents**
**Document types:** Informed consent form
**How to access documents:** glbowman@mgh.harvard.edu
**When available:** 3 months after publication and up to 5 years following article publication

**Additional Information**
**Who can access the data:** Any qualified researcher as described by study sponsor (NIH) whom has a methodologically sound proposal may gain access to data to achieve specific aims in the approved proposal.
**Types of analyses:** De-identified participant data that underlie these results (i.e., text, tables, and figures) and the study protocol are available 3 months and ending 5 years following article publication.
**Mechanisms of data availability:** Proposals should be directed to the corresponding author and those approved require signature of the Oregon Alzheimer's Disease Research Center (ADRC) data access agreement.
**Any additional restrictions:** Any qualified researcher as described by study sponsor (NIH) whom has a methodologically sound proposal may gain access to data to achieve specific aims in the approved proposal.
